# Characterizing research partnerships in child health research: A scoping review

**DOI:** 10.1177/13674935241231346

**Published:** 2024-02-06

**Authors:** Leah K Crockett, Shannon D Scott, S Michelle Driedger, Masood Khan, Devashree Prabhu, Nicole Askin, Dawn Steliga, Olivia Tefft, Ann Jansson, Sarah Turner, Kathryn M Sibley

**Affiliations:** 1 Department of Community Health Sciences, Rady Faculty of Health Sciences, 423134 University of Manitoba, Winnipeg, MB, Canada; 2 Faculty of Nursing, University of Alberta, Edmonton, AB, Canada; 3 Knowledge Translation, George & Fay Yee Centre for Healthcare Innovation, Winnipeg, MB, Canada; 4 WRHA Virtual Library, University of Manitoba, Winnipeg, MB, Canada; 5 Children’s Hospital Research Institute of Manitoba, 8664 University of Manitoba, Winnipeg, MB, Canada

**Keywords:** Capacity building, child health, children’s participation, patient participation, translational science

## Abstract

Research partnerships between researchers and knowledge users (KUs) in child health are understudied. This study examined the scope of KU engagement reported in published child health research, inclusive of health research partnership approaches and KU groups. Search strategies were developed by a health research librarian. Studies had to be in English, published since 2007, and were not excluded based on design. A two-step, multiple-person hybrid screening approach was used for study inclusion. Data on study and engagement characteristics, barriers and facilitators, and effects were extracted by one reviewer, with 10% verified by a second reviewer. Three hundred fifteen articles were included, with 243 (77.1%) published between 2019 and 2021. Community-based participatory research was the most common approach used (*n* = 122, 38.3%). Most studies (*n* = 235, 74.6%) engaged multiple KU groups (range 1–11), with children/youth, healthcare professionals, and parents/families being most frequently engaged. Reporting of barriers and facilitators and effects were variable, reported in 170 (53.8%) and 197 (62.5%) studies, respectively. Publications have increased exponentially over time. There is ongoing need to optimize evaluation and reporting consistency to facilitate growth in the field. Additional studies are needed to further our understanding of research partnerships in child health.

## Background

Collaborations between researchers and knowledge users (KUs) have advanced in recent decades to bridge evidence to practice gaps in health care and promote more relevant, meaningful, and impactful research (Kothari et al., 2017). These collaborative research approaches may include integrated knowledge translation (iKT), community-based participatory research (CBPR), and patient and public engagement (PPE)—distinct terms with often overlapping principles and constructs (Nguyen et al., 2020). In this paper we refer to these approaches collectively as *research partnerships* (Hoekstra et al., 2018), which we define as actively involving KUs in research governance and activities that leads to knowledge co-production and use (Graham and Tetroe, 2009; Jull et al., 2019). Those considered KUs can include healthcare professionals, policy makers, educators, decision makers, administrators, community members, and patients and the public, who are likely able to use knowledge generated through research to make informed decisions about health behaviors, health policies, programs, and/or practices (Canadian Institutes of Health Research, 2021a). Existing syntheses suggest that research partnerships may contribute to improved relevance, acceptance, and application of research findings, with tangible effects on health system outcomes, policy, and practice (Hoekstra et al., 2020). However, comprehensive syntheses of research focused on partnerships in child health have yet been undertaken.

Just as pediatric healthcare is distinct from adult (Klassen et al., 2008; Larcher, 2017), we propose that research partnerships in child health possess features that may be unique relative to partnerships addressing adult health. Engaging children and youth themselves requires addressing a range of developmental, ethical, and practical considerations (Clarke, 2015; Jacquez et al., 2013; Larsson et al., 2018; McLaughlin, 2006). Parents and caregivers are often important advocates for young children in research who may not be able to speak for themselves (Banner et al., 2019; Curran et al., 2018; Vaughn et al., 2013). Similarly, policy and decision makers, and school representatives often serve as proxies to support the well-being and interests of children and youth in research processes (Hamdani et al., 2021). Healthcare professionals are also important advocates for families and children (Molloy et al., 2019). These important but indirect stakeholders in child health add complexity to partnering in research (Curran et al., 2018).

Children and their families have indicated a desire for engagement in planning, designing, and implementing research projects (Ennis and Wykes, 2013), and there is evidence that engagement is increasing (Bradbury-Jones et al., 2018; Freire et al., 2022). However, many existing frameworks equate engagement with adult models of engagement and do not give special consideration to how research should be conducted with children and youth, parents and families (Woodgate et al., 2018), or healthcare professionals (Molloy et al., 2019) in child health. Further, available guidance and examples of research partnerships in child health research remain substantially smaller than that of adult work (Bradbury-Jones et al., 2018).

Existing syntheses focusing on research partnerships in child health have been limited in scope. Reviews have identified a lack of robust descriptions of strategies used to engage (Bailey et al., 2015; Flynn et al., 2019; Shen et al., 2017), limiting our knowledge of effective engagement strategies within child health. These are compounded by the predominant focus on PPE with specific KUs (e.g., parents and families, children, and youth). Although several studies have identified benefits and challenges to engaging parents (Flynn et al., 2019; Shen et al., 2017) and young people (Flynn et al., 2019; Jacquez et al., 2013; Vaughn et al., 2013) in research, evidence often relied on anecdotal and subjective accounts and limited evidence to support our understanding of their effects (Jacquez et al., 2013; Shen et al., 2017; Vaughn et al., 2013). Reviews have also noted limited evaluation and reporting of outcomes (Bailey et al., 2015, Rouncefield-Swales et al., 2021), and ambiguities in reporting quality (Freire et al., 2022). Describing factors affecting engagement can facilitate our understanding of research partnership outcomes; yet, reporting of barriers and facilitators in extant literature has been low (Freire et al., 2022).

Despite differences in terminologies, concepts, and origins among research partnership approaches, their similarities have been described (Nguyen et al., 2020), supporting need for a comprehensive review. Further, evidence suggests that researchers use of a combination of principles from multiple approaches when conducting research partnerships and report challenges in aligning with a single approach (Nguyen et al., 2020). While it is important to highlight research partnerships within specific child health contexts (e.g., disability and medical research), with specific KU groups (e.g., parents and families) or using specific research partnership approaches (e.g., CBPR), these traditionally siloed approaches limit our ability to advance broader understanding and mutual learning across research partnership approaches (Hoekstra et al., 2020).

### Aim

The aim of the study was to examine study engagement characteristics, barriers and facilitators, and effects of research partnerships in child health, inclusive of research partnership approaches and KU groups.

## Materials and methods

### Review framework

This scoping review was guided by the Arksey and O’Malley scoping review framework (Arksey and O'Malley, 2005; Levac, Colquhoun and O’Brien, 2010) which lends structure for identifying research questions and relevant studies, selecting studies, charting data, and collating, summarizing, and reporting results. Reporting of scoping review processes and findings were guided by the Preferred Reporting Items for Systematic Reviews and Meta-Analysis Extension for Scoping reviews (PRISMA-ScR) (Tricco et al., 2018). A PRISMA-ScR checklist is provided in supplemental file 1. Given noted limitations across research partnership literature, including poor indexing of various approaches (Camden et al., 2015; Gagliardi et al., 2016), this review aligns and builds on a search strategy and conceptual framework collaboratively developed by Hoekstra colleagues (2018) focusing on strategies, outcomes, and impacts of health research partnerships.

### Identifying relevant studies

A medical librarian (NA) developed and executed a search strategy comprising subject headings and free-text terms, developed collaboratively for use across a range of research partnership syntheses (Hoekstra et al., 2018), with additional constraints set to limit partnerships to the child population (from birth to 18 years). Studies were identified through a search of the following databases: Medical Literature Analysis and Retrieval Online System (MEDLINE), Cumulative Index to Nursing and Allied Health Literature (CINAHL), Excerpta Medica Database (Embase), and Psychological Information Database (PsycINFO). Searching was initially conducted in April 2019 and updated in October 2021.

### Inclusion criteria

Primary peer-reviewed studies written in English were eligible for inclusion. Studies were not excluded based on study design and included studies using quantitative, qualitative, or mixed methods. For inclusion, articles needed to describe involvement of KUs in at least one phase of research, conducting research focused on child and/or youth health. Additionally, articles needed to describe at least one aspect of study or engagement characteristics, barriers and facilitators, or effects of this partnership. Results were included dating back to 2007 for feasibility. Articles without a focus on children or youth (from birth to 18 years) or a focus on health (e.g., academic outcomes) were excluded. Studies that focused on youth, but included some participants ages 18+ (e.g., mental health), were included if child health was of predominant focus. Commentaries, protocols, reviews, and grey literature were not included.

### Study selection

After de-duplication, we screened eligible articles for inclusion. Screening was managed using Rayyan (Ouzzani et al., 2016). Level 1 screening was conducted by multiple reviewers (LKC, MK, DP, OT, DS, and ST). Each reviewer was initially assigned a random sample of 100 title and abstracts, which were screened in duplicate with the principal author (LKC), and any remaining discrepancies were resolved by discussion. Articles were then screened independently and assigned to categories of “include,” “exclude,” or “unsure.” During level II screening, full-text articles were retrieved and reviewed by multiple reviewers (LKC, MK, and DP) in a similar fashion. A second reviewer (LKC and MK) independently reviewed 10% of all articles at both levels I and II of screening to assess screening reliability.

### Data extraction and analysis

A data extraction form was developed and tested by LKC on a sample of 10 included studies and refined iteratively (Levac et al., 2010). Data extraction variables were pre-defined categorically based on existing literature (see supplemental file 2), with an “other” option, which were organized into additional categories by LKC and summarized. Data were extracted by LKC in four broad categories outlined in supplemental file 2. A second reviewer (KMS) independently extracted data from 10% of articles for quality assurance, and any discrepancies were discussed and resolved. LKC revised extraction protocols as needed per discussion, and unclear items were discussed and resolved with KMS.

Results were summarized quantitatively using descriptive statistics (frequencies and proportions) and are presented through tables and a narrative summary (Grant and Booth, 2009). Due to secondary analysis of published data, ethical permission was not sought for this review.

## Results

### Study characteristics

**Figure 1.**
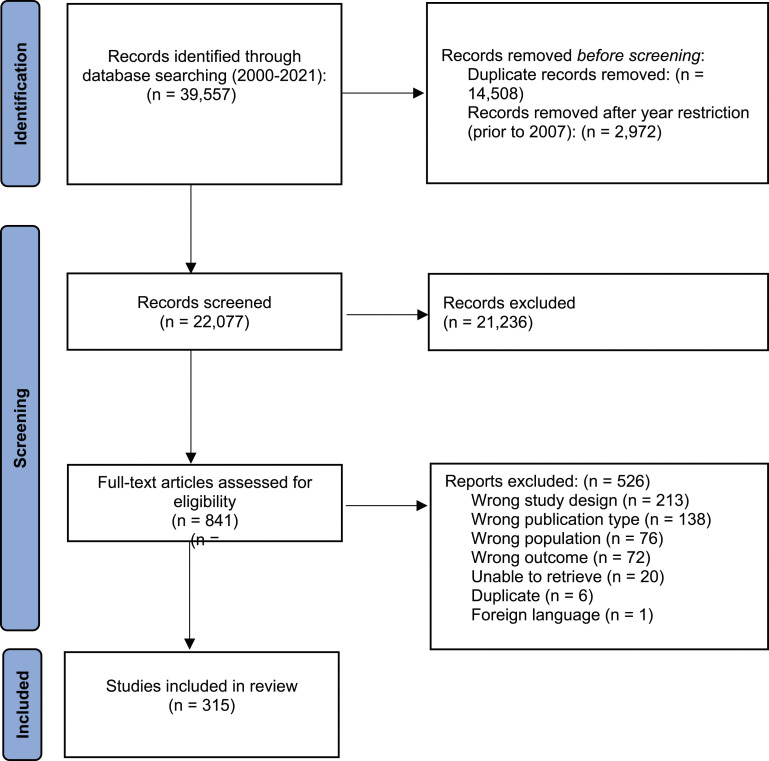
PRISMA flow diagram for a scoping review of research partnerships in child health.

Screening and selection are depicted in Figure 1. Records (n = 39,557) were identified through database searching. After deduplication and year restriction, 22,077 articles were identified for screening and 841 full-text articles were subsequently obtained for review. A total of 315 articles were included (supplemental file 3).

Study characteristics are reported in Table 1. Most articles originated from the United States (*n* = 148, 47.0%). Publication volume increased across each time period, with 243 (77.1%) published between 2019 and 2021. Most studies used mixed-methods (*n* = 128, 40.6%) research designs. Most studies address issues crossing multiple age ranges (*n* = 156, 49.7%). Research partnership approaches were described using numerous terms, with community-based participatory research (CBPR) (*n* = 122, 38.3%) used most frequently. Children or youth (*n* = 171, 54.1%) were most frequently engaged; however, most studies engaged with more than one KU group (*n* = 235, 74.4%; range: 1–11). The most common research focus was physical activity, obesity, diabetes, and nutrition (*n* = 55, 17.5%).

**Table 1.** Characteristics of studies included in a scoping review of research partnerships in child health.

**Variable**	***n*** ** (%)**
**Publication year**	
2007–2010	10 (3.2)
2011–2014	21 (6.7)
2015–2018	41 (13.0)
2019–2021	243 (77.1)
**Region of primary author**	
North America	194 (61.6)
United States (*n* = 148); Canada (*n* = 46)	
Europe	73 (23.2)
United Kingdom (*n* = 44); Netherlands (*n* = 7); Ireland (n = 4); Italy	
(*n* = 4); Denmark (*n* = 2); Finland (*n* = 2); Germany (*n* = 2); Spain (*n* = 2),	
Sweden (*n* = 2); France (*n* = 1); Austria (*n* = 1); Finland (*n* = 1); Estonia	
(*n* = 1)	
Oceania	25 (7.9)
Australia (*n* = 21); New Zealand (n = 4)	
Africa	12 (3.8)
Kenya (*n* = 5); South Africa (*n* = 3); Zimbabwe (*n* = 2); Nigeria (*n* = 1);	
Uganda (*n* = 1)	
Middle East	5 (1.6)
Israel (*n* = 4); Iran (*n* = 1)	
Asia	4 (1.3)
Thailand (*n* = 3); South Korea (*n* = 1)	
South America	2 (0.6)
Peru (*n* = 1); Columbia (*n* = 1)	
**Research design**	
Mixed methods	128 (40.6)
Qualitative	122 (38.7)
Quantitative	65 (20.7)
**Study population**	
Spanning multiple age groups	156 (49.7)
Adolescence (12–18 years)	90 (28.5)
Child (2–11 years)	35 (11.1)
Infant (0–1 years)	10 (3.1)
Unclear	24 (7.6)
**Partnership approach listed by authors**	
Community-based participatory research (CBPR)	121 (38.3)
Participatory research	37 (11.7)
Participatory action research (PAR)	31 (9.8)
Collaborative research	24 (7.9)
Co-creation/co-design/co-production	22 (7.0)
Patient engagement (PE) or patient and public engagement (PPI)	15 (4.7)
Integrated knowledge translation (iKT)	10 (3.2)
Action research (AR)	6 (1.9)
Other terms	14 (4.4)
Stakeholder engagement (*n* = 2); community-engaged research	
(*n* = 2); community-based KT (*n* = 1); academic-practice partnership	
(*n* = 1); community-academic partnership (*n* = 1); integrated	
research-practice partnership (*n* = 1); integrated participatory	
approach (*n* = 1); participatory community engagement (*n* = 1);	
researcher-community partnership (*n* = 1); community- based and	
collaborative research (*n* = 1); integrated research-practice	
partnership (*n* = 1); consumer and community involvement (*n* = 1)	
No term used	35 (11.1)
**Knowledge user groups engaged** ^a^	
Children and youth	171 (54.3)
Healthcare professional(s)	121 (38.4)
Parents and families	119 (37.8)
Community member(s)	102 (32.4)
School representative(s)	67 (21.3)
Community-based organization	63 (20.0)
Community organization representative	47 (14.9)
Healthcare manager or administrator	43 (13.6)
Government representative	40 (12.7)
Municipal/provincial/state health agencies	30 (9.5)
Indigenous representative(s) (e.g., elders)	30 (9.5)
Social services (e.g., justice)	19 (6.0)
Non-governmental organization (NGO)	19 (6.0)
Funding organization representative	7 (2.2)
Faith-based representative(s)	5 (1.6)
**Multiple knowledge user groups engaged**	
Yes	235 (74.6)
No	80 (25.4)
**Number of knowledge user groups engaged**	
1	80 (25.4)
2	82 (26.0)
3	78 (24.8)
4	27 (8.6)
5+	48 (15.2)
**Research focus**	
Physical activity, obesity, diabetes, and nutrition	55 (17.5)
Pediatric subspecialties (e.g., acute care, cancer care, and rehabilitation)	54 (17.1)
General health (e.g., chronic pain, well-being, and sleep)	50 (15.9)
Mental health	39 (12.4)
Public health (e.g., health promotion, immunization, and injury prevention)	36 (11.4)
Sexual and reproductive health (e.g., HIV, STI, and pregnancy)	34 (10.8)
Substance use and addiction	21 (6.6)
Health services	20 (6.3)
Youth violence	6 (1.9)

^a^more than one option could be selected; percentages do no equal 100%.

### Engagement characteristics

Engagement characteristics are summarized in Table 2. KUs were most commonly engaged at the IAP2 level of *involve (i.e., working directly through the process to ensure that concerns and aspirations are consistently understood or considered *(International Association for Public Participation, 2018)*)* (*n* = 129, 41.0%). Engagement was documented at every stage of the research process, most often in *conducting* (*n* = 258, 81.6%) research. KUs were most frequently engaged in data collection (*n* = 205, 65.1%), developing study design and methods (*n* = 188, 59.7%), and data analysis and interpretation (*n* = 170, 59.7%), and least in research ethics development (*n* = 47, 14.9%). Numerous strategies (range: 1–10 per article) were reported for engaging with KUs, with formal meetings being most frequent (*n* = 261, 82.9%). However, most (*n* = 227, 72.1%) reported using multiple strategies to engage (mean = 3.42, SD = 2.67). Thirty-nine studies (12.4%) did not report on strategies used to engage.

**Table 2.** Engagement characteristics for KU involvement in child health research partnerships.

**Variable**	***n*** ** (%)**
**Level of engagement**	
Consult	68 (21.6)
Involve	129 (41.0)
Collaborate	106 (33.6)
Empower	12 (3.8)
**Research stage**	
Planning	238 (75.3)
Conducting	258 (81.6)
Disseminating	133 (42.1)
**Research activities**	
Data collection	205 (65.1)
Developing study design and methods	188 (59.7)
Data analysis and interpretation	170 (54.0)
Choosing study outcomes	140 (44.4)
Setting research priorities	125 (39.7)
Choosing research questions	124 (39.4)
Dissemination to non-academic audiences	102 (32.4)
Participant recruitment	102 (32.4)
Dissemination to academic audiences	62 (19.7)
Development of research ethics	47 (14.9)
**Strategies for engaging**	
Formal meetings	261 (82.9)
Establishment of formal working groups	140 (44.4)
Formal updates	118 (37.5)
Distribution of study documents	116 (36.8)
Provision of training opportunities or resource materials	113 (35.9)
Informal conversations	105 (33.3)
Development of formal documentation of processes	84 (26.7)
Honorariums for KUs	38 (12.1)
Researchers attending KU meetings or events	39 (12.4)
Provision of social opportunities	37 (11.7)
Shared electronic space	25 (7.9)
Sharing of research funds with KUs	4 (1.3)
Not specified	39 (12.4)
**Evaluated partnership**	
Yes	98 (31.1)
No	217 (68.9)

### Barriers and facilitators

Barriers and facilitators were reported in just over half of included studies (*n* = 170, 53.8%) and are reported in Table 3. Most frequently reported facilitators of KU engagement included maintaining good communication between researchers and KUs (*n* = 107, 34.0%) and having clearly defined roles and expectations (*n* = 106, 33.7%). The most frequently reported barrier was time (*n* = 29, 9.2%).

**Table 3.** Barriers and facilitators of engaging in research partnerships in child health.

**Barriers and facilitators reported**		***n*** ** (%)**
	Yes	170 (53.8%)
	No	145 (46.2%)

**FACILITATORS**	**Description**	***n*** ** (%)**
Communication	Maintaining good communication between researchers and KUs	107 (34.0%)
Roles and expectations	Clearly defined roles and expectations	106 (33.7%)
Facilitation	Involving professional facilitators or knowledge brokers to support researchers and/or KUs	97 (30.8%)
Capacity (resources)	Having sufficient infrastructure and resources to conduct research partnerships	80 (25.4%)
Valuing expertise	Equitable engagement, including valuing all knowledge, experiences, and perspectives	79 (25.1%)
Skills	Having expertise and skills to conduct research partnerships	69 (21.9%)
Alignment	Having aligned goals, objectives, and priorities between researchers and KUs	68 (21.6%)
Opportunities	Having training and education opportunities	56 (17.8%)
Cultural considerations	Attention and alignment to cultural values, customs, and priorities	54 (17.1%)
Commitment	Having commitment, buy in, and support for the project; researchers attending events without research-specific activities	44 (14.0%)
Fostering relationships	Spending time and effort to build and nurture trustful, respectful relationships	31 (9.8%)
Compensation	Compensation of partners for their time and accommodating needs	26 (8.3%)
Pre-existing relationships	Having pre-existing/established relationships between researchers and KUs	18 (5.7%)
Ongoing partnership	Ongoing collaboration between researchers and KUs beyond the research project	11 (3.2%)
Early partnership	Establishing partnerships early in the research process	10 (3.2%)
Technology	Leveraging e-technology	9 (2.9%)
Flexibility	Accommodating schedules, allowing time, and flexibility within research agreements	6 (1.9%)
Power differentials	Awareness and attention to power differentials	5 (1.6%)
Shared governance	Having shared governance/decision-making structures	4 (1.3%)
**BARRIERS**		
Time	Having time constraints and time required to build relationships	29 (9.2%)
Competing agendas, interests, and priorities	Competing agendas, interests, and priorities	25 (7.9%)
Capacity (resources)	Having a lack of resources to support research partnerships	19 (6.0%)
Power imbalances	Managing power and group dynamics	13 (4.1%)
Retention	Frequent turnover of KUs and/or ongoing organizational/leadership change	10 (3.2%)
Inconsistent engagement	Inconsistent or unequal engagement of KUs	9 (2.9%)
Geography	Having a large geographical distance between researchers and KUs	9 (2.9%)
Skills	Lack of skills, expertise, and/or capacity of researchers or KUs to engage in research partnerships	7 (2.2%)
Institutional and funding	Limitations or challenges imposed by institutional regulations or grant funding timelines or regulations	6 (1.9%)
Attendance	Meetings poorly attended	6 (1.9%)
Unclear goals, roles, and expectations	Having unclear goals, roles, and expectations	4 (1.3%)
Health challenges	Competing or existing health challenges among team members	3 (1.0%)

### Effects

Effects of engaging KUs within the research process were reported in 197 (62.5%) studies. Of articles reporting effects, all (*n* = 197, 100.0%) reported beneficial effects while only 15 (4.8%) reported effects that were challenging. The most frequent beneficial effects were related to the research process, including developing relevant and useful research findings (*n* = 125, 39.7%) and creating high-quality research, such as generating new projects (*n* = 111, 35.2%). While few reported challenging effects of research partnerships (*n* = 15, 4.8%), the most common were at the partnership level including conflicts between researchers and KUs (*n* = 14, 4.4%). Further details are provided in Table 4 and full definitions can be found in supplemental file 4.

**Table 4.** Effects of health research partnerships reported in included studies.

**Effects reported**	**n (%)**
Yes	197 (62.5%)
No	118 (37.5%)

**Subcategory**	**Beneficial effects**	***n*** ** (%)**	**Challenging effects**	***n*** ** (%)**
**INDIVIDUAL LEVEL**				
***Effects on researchers conducting partnership research***	Increased capacity, knowledge, and skills	92 (29.2%)	Personal challenges	11 (3.5%)
	Personal benefits	24 (7.6%)		
***Effects on KUs involved in research partnerships***	Increased “capacity, knowledge, and skills”	100 (31.7%)	Personal challenges	5 (1.6%)
	“Positive attitude” towards research and researchers	27 (8.6%)	Feeling overburdened by tasks and responsibilities	1 (0.3%)
	Better access to information	71 (22.5%)		
	Personal benefits	64 (20.3%)		
**PARTNERSHIP LEVEL**				
***Effects on the relationship between researchers and KUs (partnership level)***	Positive outcomes/impacts on the relationship between researchers and KUs	86 (27.3%)	Conflicts between researchers and KUs	14 (4.4%)
**COMMUNITY OR SOCIETY LEVEL**				
***Effects on the community or society***	Increased acceptability and trust of the research	80 (25.4%)	Challenging outcomes or impacts on the community	0 (0.0%)
	Increased capacity in the community	79 (25.1%)		
	Increased community ownership of the research	52 (16.5%)		
	Creating system changes or action	50 (15.9%)		
	Increased community empowerment	35 (11.1%)		
**RESEARCH PROCESS LEVEL**				
***Effects on the research process***	Creating “relevant and useful research findings”	125 (39.7%)	Biased data or tokenism	8 (2.5%)
	Creating “high-quality research”	111 (35.2%)		
	Increased capacity to conduct and disseminate research	79 (25.1%)		

## Discussion

This scoping review examined study engagement characteristics, barriers and facilitators, and effects of research partnerships in child health, inclusive of research partnership approaches and KU groups. We highlight four findings of interest: (1) exponential growth in publications reporting research partnerships in child health over time, with primary origins from the United States, Canada, the United Kingdom, and Australia; (2) predominant reporting of CBPR as an approach within included studies; (3) engagement of multiple KU groups at varying levels of engagement; and (4) variable and selective reporting of facilitators and positive effects of engaging.

Data indicate a doubling in publications on child health research partnerships across each time period, with a five-fold increase between 2015–2018 and 2019–2021. Our findings suggest that research partnerships in child health are increasing, regardless of approach or geographical region, though most publications originate from the United States, Canada, the United Kingdom, and Australia. Growth in publication over time predominating mainly from these countries may be reflective of targeted funder-driven efforts to encourage and promote research partnerships to maximize impacts of research investment (Canadian Institutes of Health Research, 2015; Canadian Institutes of Health Research, 2021a; Rycroft-Malone et al., 2011). Other studies have indicated an increase in published literature using participatory approaches with children and youth (Bradbury-Jones et al., 2018). Similar to Hoekstra et al. (2020), we observed differences in approaches based on origin of the primary author and by time period. For example, all studies using an iKT approach originated from Canada and were published from 2016 onwards. This likely relates to iKTs’ origins as a funder-driven approach advanced by the federal health research funding agency, the Canadian Institutes of Health Research (CIHR) (Nguyen et al., 2020). In contrast, studies using a PPE approach were more evenly distributed across countries, and studies using a CBPR approach were present across all time periods and geographical locations.

CBPR was the most frequently identified approach within the corpus of included studies. Frequent reported use of CBPR as an approach in child health may be noteworthy given its’ theoretical differences relative to other research partnership approaches (Jull et al., 2017; Nguyen et al., 2020). Due to its’ long-standing history as a research approach aimed at promoting social justice, enhancing KU skills and capacity, and use of specific methods common among CBPR methodology (e.g., digital storytelling, photovoice, focus groups, and interviews), it is possible that research traditions underlying CBPR have perhaps been historically more fitting for research partnerships in child health. Furthermore, CBPR focuses on conducting research in partnership with historically vulnerable or disenfranchised communities (Olshanksy and Zender, 2008). Alternatively, approaches such as iKT that focus on increasing knowledge use and impact do not explicitly focus on capacity building and attention to power relations (Jull et al., 2017), aspects important when engaging children and youth in particular, where power relations are heightened (Bradbury-Jones et al., 2018).

Given the complexity of health systems, Jull and colleagues (2017) argue that achieving conceptual clarity of various collaborative research approaches will allow research partnership teams to better leverage and use knowledge based on what they hope to achieve. Furthermore, due to their successes and unique strengths they urge consideration of various research partnership approaches and processes when designing and conducting collaborative research (Jull et al., 2017). We encourage researchers to be clearer and more intentional in their use of research partnership approaches to avoid epistemological and ontological slippage.

We also found that 74.6% of studies engaged with multiple KU groups, often at varying levels of engagement, suggesting that a range of individuals are involved in child health research partnerships. For example, Anang et al. (2019) first initiated discussion and relationship building with the local health committee and council in 2015, leading to the initial study planning and engagement with an Elder and school representatives. Subsequently, they engaged youth, who co-facilitated focus groups and assisted in interpretation in 2017. In this study, adult KUs took an oversight role and contributed largely to project initiation. Alternatively, Crudington et al. (2020) concurrently consulted both parents and children to obtain feedback in developing patient-oriented outcome measures (PROMs) for childhood epilepsy. This suggests varying relational, time, and resource-intensive strategies may be required to undertake research partnerships in child health, depending on project aims. The presence of adult KU groups alongside studies engaging children and youth also suggests that additional protective and safeguard strategies may be used, or required, in the context of child health. However, studies did not consistently differentiate which strategies were used with which KU group, nor was intensity of engagement by KU group clearly reported, suggesting a need for enhanced clarity in reporting.

While KUs were engaged at varying levels of intensity, 74.6% of studies engaged at levels of involve or collaborate, suggesting moderate to high levels of engagement. Several studies suggested need for flexible levels of engagement among KUs within teams. For example, Funk et al. (2012) noted that although their team climbed Hart’s ladder of participation (i.e., a model of participation contextualized to young people’s participation; Hart, 1992) as the project progressed, not all youth climbed at similar speeds or participated at similar levels based on their interest, strengths, and abilities. Similarly, Van Staa et al. (2010) suggested that level of participation in the research process should be negotiated as equitable rather than equal, negotiated based on preferences and interests of KUs. This corresponds to the practicality principle of proportional involvement, as outlined by Liabo et al. (2020) which involves balancing involvement throughout the research process based on individual demands and project resources and supports.

Like existing reviews, reporting of barriers and facilitators and effects were variable, reported in 53.8% and 62.5% of studies, respectively. Similarly, reporting of effects was largely anecdotal, based on perceived subjective accounts of authors. This adds to the growing body of evidence suggesting a need for more systematic evaluation and measurement of effects in research partnerships in child health (Vaughn et al., 2013; Freire et al., 2022; Rouncefield-Swales et al., 2021; Bradbury-Jones et al., 2018). Most notably, reporting was heavily weighted towards the positive aspects of engaging, suggesting biased reporting and limiting our ability to constructively learn from the challenges faced by research teams engaging in research partnerships. Van Staa et al. (2010) noted that although not all partnerships lead to tangible or positive effects, challenges are not typically reported. Hoekstra et al. (2020) also noted this predominant reporting of beneficial effects of research partnerships. However, as highlighted by Kothari and Wathen (2013), the process itself is often more beneficial than the outcomes (Van Staa et al., 2010). It is important to note that researchers are not likely to have reported on a comprehensive list of effects depending on study purpose. However, it can be hypothesized that those reported reflect those most prominently experienced, or of those aspects agreed upon for reporting within the partnership engagement process itself. Leveraging prominent facilitators identified in this review may serve to mitigate challenges encountered by research teams.

### Limitations

Our review was limited to studies published in 2007 and later for feasibility and relevant studies dating back to 2000 were minimal. Limiting our review to studies published in English may bias results to developed countries. Electronic literature searches to identify research partnerships often lack precision and require considerable time and resources. Emerging methods such as semi-automated text mining for title and abstract screening could be explored to facilitate this process (Pham et al., 2021). We identified that use of terminology does not necessarily reflect a true partnership and that there may be varying degrees of conceptual understanding across fields. Others have noted a similar trend, with terms sometimes used erroneously to indicate research that is merely conducted *on* people versus *with* people (Vaughn et al., 2013). Due to variable reporting of engagement characteristics within studies and challenges in clearly delineating which strategies were used with which KUs, we were unable to examine differences by KU group. There is potential that relevant articles were excluded in title and abstract screening if they did not make reference of research partnerships in the abstract. However, we minimized the risk of missing papers as much as possible by advancing any paper that was unclear regarding eligibility in the abstract screening phase for full-text screen. Finally, for similar feasibility reasons, we also chose to exclude studies which may have indirect effects on child health (e.g., academic performance).

### Implications

Although the co-existence of multiple research partnership approaches is well-recognized, these approaches have remained largely disconnected in the literature. This hampers learning across approaches in designing, conducting, assessing, and determining effects of health research partnerships. Due to broad vocabulary used to describe health research partnerships and variable levels of reporting across approaches, there remains a need to provide reporting guidance to facilitate ongoing efforts (Boland et al., 2020). However, there are limitations in partnership-based reporting. For example, while conversations regarding challenges encountered may occur internally, they are not necessarily shared externally in peer-reviewed publications for a multitude of reasons.

By leveraging common search strategies terminology outlined by a multi-coordinated team (Hoekstra et al., 2018), this study begins to optimize research quality and consistency across reviews and provides a baseline view of research partnerships conducted in child health. Researchers looking for reporting guidance may look to Hoekstra et al. (2020) to improve consistency. Further, pre-emptive evaluation planning can serve to enhance our understanding of partnership effects.

The findings of this review have practical implications for a broad audience. This review provides insight for both researchers and KUs conducting research in a partnered way in the child health sphere by outlining when and how various KUs may be engaged in the research process and with what effect.

## Conclusion

This review is unique in scope as we synthesized literature in child health across partnership approaches and KU groups. We note exponential growth in research partnerships in child health, particularly since 2019, and predominance of CBPR as a research partnership approach in child health. Findings highlight variable levels of reporting of engagement characteristics, barriers and facilitators, and effects within the published literature, suggesting need for guidance to support evaluation and consistent reporting. Further, engagement of multiple KU groups at varying intensities within studies introduces challenges in differentiating engagement characteristics within and between studies, and it is not always evident how KUs are engaged in the research process or if there are approaches unique to child health. However, this review begins to outline diverse research partnership literature in child health, providing a starting point to enhance our understanding of practices used within child health. Further research is needed to better understand principles and relational aspects that may render child health a unique context for research partnerships.

## Supplemental Material


Supplemental Material - Characterizing research partnerships in child health research: A scoping review
Supplemental Material for Characterizing research partnerships in child health research: A scoping review by Leah K Crockett, Shannon D Scott, S Michelle Driedger, Masood Khan, Devashree Prabhu, Nicole Askin, Dawn Steliga, Olivia Tefft, Ann Jansson, Sarah Turner, and Kathryn M Sibley in Journal of Child Health Care.

## Data Availability

All reported data are included in the manuscript and supplementary materials. Further data are available from the corresponding author on reasonable request.
