# Supplementary material for: Characterizing research partnerships in child health research: A scoping review
Source: J Child Health Care. 2024 Feb 6;29(3):698–715. doi: 10.1177/13674935241231346 (PMC12368297; doi:10.1177/13674935241231346)
Supplement: Supplemental Material - Characterizing research partnerships in child health research: A scoping review [file sj-pdf-1-chc-10.1177_13674935241231346.pdf]

Supplementary file 1. Preferred Reporting Items for Systematic reviews and Meta-Analyses extension for Scoping Reviews (PRISMA-ScR) Checklist for "Characterizing research partnerships in child health: a scoping review"

SECTION	ITEM	PRISMA-ScR CHECKLIST ITEM	REPORTED ON PAGE #
**TITLE**			
Title	1	Identify the report as a scoping review.	1
**ABSTRACT**			
Structured summary	2	Provide a structured summary that includes (as applicable): background, objectives, eligibility criteria, sources of evidence, charting methods, results, and conclusions that relate to the review questions and objectives.	1
**INTRODUCTION**			
Rationale	3	Describe the rationale for the review in the context of what is already known. Explain why the review questions/objectives lend themselves to a scoping review approach.	2-4
Objectives	4	Provide an explicit statement of the questions and objectives being addressed with reference to their key elements (e.g., population or participants, concepts, and context) or other relevant key elements used to conceptualize the review questions and/or objectives.	4-5
**METHODS**			
Protocol and registration	5	Indicate whether a review protocol exists; state if and where it can be accessed (e.g., a Web address); and if available, provide registration information, including the registration number.	N/A
Eligibility criteria	6	Specify characteristics of the sources of evidence used as eligibility criteria (e.g., years considered, language, and publication status), and provide a rationale.	6
Information sources*	7	Describe all information sources in the search (e.g., databases with dates of coverage and contact with authors to identify additional sources), as well as the date the most recent search was executed.	6
Search	8	Present the full electronic search strategy for at least 1 database, including any limits used, such that it could be repeated.	N/A
Selection of sources of evidence†	9	State the process for selecting sources of evidence (i.e., screening and eligibility) included in the scoping review.	6-7
Data charting process‡	10	Describe the methods of charting data from the included sources of evidence (e.g., calibrated forms or forms that have been tested by the team before their use, and whether data charting was done independently or in duplicate) and any processes for obtaining and confirming data from investigators.	7
Data items	11	List and define all variables for which data were sought and any assumptions and simplifications made.	Table 1
Critical appraisal of individual sources of evidence§	12	If done, provide a rationale for conducting a critical appraisal of included sources of evidence; describe the methods used and how this information was used in any data synthesis (if appropriate).	N/A
Synthesis of results	13	Describe the methods of handling and summarizing the	7

St. Michael's
Inspired Care.
Inspiring Science.

SECTION	ITEM	PRISMA-ScR CHECKLIST ITEM	REPORTED ON PAGE #
		data that were charted.	
**RESULTS**			
Selection of sources of evidence	14	Give numbers of sources of evidence screened, assessed for eligibility, and included in the review, with reasons for exclusions at each stage, ideally using a flow diagram.	7
Characteristics of sources of evidence	15	For each source of evidence, present characteristics for which data were charted and provide the citations.	7-9
Critical appraisal within sources of evidence	16	If done, present data on critical appraisal of included sources of evidence (see item 12).	N/A
Results of individual sources of evidence	17	For each included source of evidence, present the relevant data that were charted that relate to the review questions and objectives.	7-9
Synthesis of results	18	Summarize and/or present the charting results as they relate to the review questions and objectives.	7-9
**DISCUSSION**			
Summary of evidence	19	Summarize the main results (including an overview of concepts, themes, and types of evidence available), link to the review questions and objectives, and consider the relevance to key groups.	10-14
Limitations	20	Discuss the limitations of the scoping review process.	14-15
Conclusions	21	Provide a general interpretation of the results with respect to the review questions and objectives, as well as potential implications and/or next steps.	15-16
**FUNDING**			
Funding	22	Describe sources of funding for the included sources of evidence, as well as sources of funding for the scoping review. Describe the role of the funders of the scoping review.	16

JBI = Joanna Briggs Institute; PRISMA-ScR = Preferred Reporting Items for Systematic reviews and Meta-Analyses extension for Scoping Reviews.
* Where *sources of evidence* (see second footnote) are compiled from, such as bibliographic databases, social media platforms, and Web sites.
† A more inclusive/heterogeneous term used to account for the different types of evidence or data sources (e.g., quantitative and/or qualitative research, expert opinion, and policy documents) that may be eligible in a scoping review as opposed to only studies. This is not to be confused with *information sources* (see first footnote).
‡ The frameworks by Arksey and O'Malley (6) and Levac and colleagues (7) and the JBI guidance (4, 5) refer to the process of data extraction in a scoping review as data charting.
§ The process of systematically examining research evidence to assess its validity, results, and relevance before using it to inform a decision. This term is used for items 12 and 19 instead of "risk of bias" (which is more applicable to systematic reviews of interventions) to include and acknowledge the various sources of evidence that may be used in a scoping review (e.g., quantitative and/or qualitative research, expert opinion, and policy document).

From: Tricco AC, Lillie E, Zarin W, O'Brien KK, Colquhoun H, Levac D, et al. PRISMA Extension for Scoping Reviews (PRISMAScR): Checklist and Explanation. Ann Intern Med. 2018;169:467–473. doi: 10.7326/M18-0850.

Supplementary file 2. Description of data extraction variables for a scoping review on research partnerships in child health

Variable	Description	Variable type	References
**Study characteristics**			
Year	Year of publication	Multiple choice	N/A
Country	Country of corresponding author	Multiple choice	N/A
Research design	Broad study design used to conduct research in partnership with researchers and KUs. Research design was classified as quantitative, qualitative, multi or mixed methods.	Multiple choice	N/A
Study population	Study population of research focus. Study population was classified as multiple (e.g., spanning multiple age ranges), infant (ages 0-1), child (ages 2-11), adolescent (ages 12-17), or unclear.	Multiple choice	(Public Health Ontario, 2013)
Research focus	Focus of research study. Study topic area was described in open ended format for the first 75 articles, then classified thematically. Categories were used for all subsequent data extraction.	Open ended; multiple choice	N/A
Partnership terminology	Terminology used to describe the partnership. Categories were pre-defined based on existing literature as: IKT, CBPR, PPE, participatory action research, participatory research, action research, collaborative research, co-production, and other.	Multiple choice	Hoekstra et al., 2020
KU groups engaged	KUs engaged within the research process. Categories were pre-defined based on existing literature as: parents and families, children and youth, healthcare professionals, community members, school representatives, community-based organizations, healthcare manager or administrator, funding organization representative, and other.	Select all	Hoekstra et al., 2020; Flynn et al., 2019
Multiple KU groups	More than one knowledge user group engaged within the research process.	Binary	N/A
Number of KU groups engaged	Number of knowledge user groups engaged within the research process. This variable was a sum of the variable 'knowledge user groups engaged'.	Numerical	N/A
Primary KU group	Where more than 1 knowledge user was engaged, the knowledge user group of prominent focus or most actively engaged within the project. Categories were pre-defined, as per variable 'KUs engaged.'	Multiple choice	Hoekstra et al., 2020; Flynn et al., 2019
**Engagement characteristics**			
Level of engagement	Level of engagement of KUs within the research process, based on the following definitions: consult (*to obtain feedback on analysis, alternatives, and/or decisions*); involve (*to work directly through the process to ensure that concerns and aspirations are consistently understood or considered*); collaborate (*to partner in each aspect of the decision including development of alternatives and identification of preferred solutions*), and empower (*to place final decision making power in*	Multiple choice	International Association of Public Participation [IAP2], 2018

	the hands of KUs; partnership initiated by KUs). Each article was classified based on the authors' report of engagement and its alignment with the above noted definitions.		
Research stage	Engagement of KUs in the planning, conduct and dissemination of research. Categories were pre-defined as: planning, conducting, and dissemination.	Select all	
Research activities	Research activities in which KUs were engaged. Categories were pre-defined as: setting research priorities, choosing research questions, developing study design and methods, development of research ethics documents, participant recruitment, data collection, data analysis and interpretation, dissemination to academic audiences, and dissemination to non-academic audiences.	Select all	Hoekstra et al., 2020
Engagement strategies	Strategies used to engage KUs in the research process. Categories were predefined as: formal meetings, establishment of formal working groups, formal updates, distribution of study documents, provision of training opportunities or resource materials, informal conversations, development of formal documentation of processes, honorariums for KUs, researchers attending knowledge user meetings or events, provision of social opportunities, shared electronic space, sharing of research funds with KUs, and not specified.	Select all	Hoekstra et al., 2020
Evaluation	Evaluation of the research partnership before, during or after the research partnership.	Binary	
**Barriers and facilitators**			
Barriers and Facilitators	Barriers and facilitators of engaging KUs in the research process. Barriers and facilitators were extracted and categorized based on known barriers and facilitators reported in research partnership literature. Initial categories were pre-defined and refined iteratively throughout the data extraction phase for all data categorized as "other".	Select all	Bird et al., 2020; Brush et al., 2020; Anna R. Gagliardi et al., 2016; Anna R Gagliardi & Dobrow, 2016; Hofmeyer, Scott, & Lagendyk, 2012; Kendall et al., 2018; Lawrence et al., 2019; Mitton, Adair, McKenzie, Patten, & Perry, 2007; Shannon L Sibbald et al., 2014)
**Effects**			

St. Michael's
Inspired Care.
Inspiring Science.

Beneficial and challenging effects	Beneficial and challenging effects of the research partnership at the individual, society and community, research partnership, and research process subcategory levels. Variable options were categorized based on the taxonomy proposed by Hoekstra et al. (2020) of effects on: 1) researchers conducting partnership research, 2) knowledge users involved in research partnerships, 3) the relationship between researchers and knowledge users, 4) the community and/or society, and 5) the research process. The term 'effects' was chosen due to poor differentiation of outcomes and impacts in the published literature (Hoekstra et al., 2020). Full definitions are provided in appendix B.	Select all	Hoekstra et al., 2020

Supplementary file 3: Definitions of research partnership effects used in scoping review of research partnerships in child health

Data extraction variables: Effects (Hoekstra et al., 2020)		
Subcategory	Beneficial Effects	Challenging Effects
	Individual level	
Effects on researchers conducting partnership research	Researchers have experienced increased **'capacity, knowledge and skills'** related to planning, conducting and disseminating research in partnership with KUs; this may include a better understanding of the area under study and/or an increased awareness of community issues	Researchers have experienced **'personal challenges'** when working in a research partnership such as uncomfortable feelings when sharing power over the research and/or the additional time and financial burden associated with the research partnership.
	Researchers have experienced **'personal benefits'** from working in a research partnership such as enhanced motivation for the research project and/or lightening of the workload	
Effects on KUs involved in research partnerships	KUs have experienced increased **'capacity, knowledge and skills'** related to research processes; this may include a better understanding of the area under study and/or an increased awareness to the application of the research	KUs have experienced **'personal challenges'** when working in a research partnership, such as feelings of not being listened to, not being empowered, not being taken seriously, frustrated and/or dissatisfied about the research processes
	KUs have experienced a more **'positive attitude'** towards research and researchers	KUs have experienced **'feeling overburdened'** by tasks and responsibilities
	KUs have reported **better access to information** relevant for them such as information on treatments or management of specific diseases or illnesses	
	KUs have experienced **'personal benefits'**	

St. Michael's
Inspired Care.
Inspiring Science.

	from working in a research partnership; examples include but are not limited to, feeling empowered, feeling valued, increased confidence, increased sense of accomplishment, extended social and support network, and/or increased chances on future employment	
Partnership level		
Effects on the relationship between researchers and KUs (partnership level)	Partners have reported that the research partnership can **'have positive outcomes/impacts on the relationship'** between researchers and KUs; examples include, but are not limited to, greater partnership synergy, mutual respect, mutual understanding of work style, language, needs and constraints, and/or can create sustainable partnerships	Partners have reported that the research partnership may result in **'conflicts'** between researchers and KUs
Community or society level		
Effects on the community or society	Partners have reported that the research partnership can **'create system changes or action'** by influencing policy-making, improving community services, improving health-related outcomes for community, and/or creating capacity to sustain the projects	Partners have reported that research partnership may create **'challenging outcomes or impacts on the community'** such as increased time and financial burden on the community organizations, further stigmatization of the group and/or negative research findings
	Partners have reported that the research partnership can **'increase capacity'** in the community by creating better understanding of research in the community and/or increased awareness and knowledge of the study topic	
	Partners have reported that the research partnership can increase **'community empowerment'**	
	Partners have reported that the research partnership can **'create community ownership'** of the research	
	Partners have reported that the research partnership can increase the **'acceptability and trust of the research'** in the community	
Research process level		
Effects on the research process	Partners have reported that the research partnership can create **'relevant and useful research findings'**	Partners have reported that the research partnership may lead to negative outcomes or impacts, including **biased data or tokenism**.
	Partners have reported that the research	

	partnership can create **'high quality research'** by generating credible and valid data, developing effective interventions, and/or unearthing new information; the partnership can also generate new and other projects	
	Partners have reported that the research partnership can create '**increased capacity'** to conduct and disseminate the research	

Supplementary file 4: Included articles

ABMA, T., LIPS, S. & SCHRIJVER, J. 2020. Sowing seeds to harvest healthier adults: The working principles and impact of participatory health research with children in a primary school context. *International Journal of Environmental Research and Public Health,* 17**,** 451.

ABRACZINSKAS, M. & ZARRETT, N. 2020. Youth Participatory Action Research for Health Equity: Increasing Youth Empowerment and Decreasing Physical Activity Access Inequities in Under-resourced Programs and Schools. *American Journal of Community Psychology,* 66**,** 232-243.

AGENOR, M., QURESHI, F., JOHNSON, K., KACEVICH, S., MCADAMS-MAHMOUD, A. & CURLIN, J. 2018. Developing a Community-Academic Partnership to Promote Mental Health Among Underserved Preadolescent Girls in Boston, Massachusetts. *Prog Community Health Partnersh,* 12**,** 321-328.

AGHAZADEH, S. A., ALDOORY, L. & MILLS, T. 2020. Integrating Health Literacy Into Core Curriculum: A Teacher-Driven Pilot Initiative for Second Graders. *The Journal of school health,* 90**,** 585-593.

AKWATAGHIBE, N. N., OGUNSOLA, E. A., POPOOLA, O. A., AGBO, A. I. & DIELEMAN, M. A. 2021. Using participatory action research to improve immunization utilization in areas with pockets of unimmunized children in Nigeria. *Health Research Policy & Systems,* 19**,** 1-14.

ALDISS, S., WHELAN, J. S., FERN, L. A., PHILLIPS, R. S., CALLAGHAN, A., HAMRANG, L., MCGEACHY, D., VEITCH, L., WILLIAMSON, M., DYKER, K., GRAVESTOCK, H., GROSZMANN, M., HOUGH, R., MORGAN, S., SMITH, S., VEITCH, H., UPADHYAYA, S. & GIBSON, F. 2019. Research priorities for young people with cancer: A UK priority setting partnership with the James Lind Alliance. *BMJ Open,* 9**,** e028119.

ALESSANDRINI, E., BRAKO, F., SCARPA, M., LUPO, M., BONIFAZI, D., PIGNATARO, V., CAVALLO, M., CULLUFE, O., ENACHE, C., NAFRIA, B., CLAVEROL, J., DE TAEYE, L., VERMEULEN, E., PRESTON, J. & TULEU, C. 2021. Children's Preferences for Oral Dosage Forms and Their Involvement in Formulation Research via EPTRI (European Paediatric Translational Research Infrastructure). *Pharmaceutics,* 13.

ALEXANDER, R., ESTABROOKS, P., BROCK, D.-J. P., HILL, J. L., WHITT-GLOVER, M. C. & ZOELLNER, J. 2021. Capacity Development and Evaluation of a Parent Advisory Team Engaged in Childhood Obesity Research. *Health promotion practice,* 22**,** 102-111.

ALI, S., DE VIGGIANI, N., ABZHAPAROVA, A., SALMON, D. & GRAY, S. 2020. Exploring young people's interpretations of female genital mutilation in the UK using a community-based participatory research approach. *BMC Public Health,* 20**,** 1-15.

AMSDEN, J. & VANWYNSBERGHE, R. 2016. Community mapping as a research tool with youth. *Action Research,* 3**,** 357-381.

ANANG, P., NAUJAAT ELDER, E. H., GORDON, E., GOTTLIEB, N. & BRONSON, M. 2019. Building on strengths in Naujaat: the process of engaging Inuit youth in suicide prevention. *International journal of circumpolar health,* 78**,** 1508321.

ANDERSON, J. B., BROWN, D. W., LIHN, S., MANGEOT, C., BATES, K. E., VAN BERGEN, A. H., RUDD, N. A., HANKE, S., TWEDDELL, J. & LANNON, C. 2019. Power of a Learning Network in Congenital Heart Disease. *World journal for pediatric & congenital heart surgery,* 10**,** 66-71.

ARMSTRONG, L. L., WATT, E., ST. JOHN, E. & DESSON, S. 2020. The interactive symptoms assessment: I.S.A. - development and validation using a knowledge translation-integrated model. *Current Psychology: A Journal for Diverse Perspectives on Diverse Psychological Issues***,** No-Specified.

ARNOLD, T. J., MALKI, A., LEYVA, J., IBARRA, J., DANIEL, S. S., BALLARD, P. J., SANDBERG, J. C., QUANDT, S. A. & ARCURY, T. A. 2019. Engaging Youth Advocates in Community-Based Participatory Research on Child Farmworker Health in North Carolina. *Progress in community health partnerships : research, education, and action,* 13**,** 191-199.

ARONOWITZ, T. & ECHE, I. 2013. Parenting strategies African American mothers employ to decrease sexual risk behaviors in their early adolescent daughters. *Public Health Nurs,* 30**,** 279-87.

BAKAKI, P. M., STALEY, J., LIU, R., DAWSON, N., GOLCHIN, N., HORACE, A., JOHNSON, H., WALDRON, J., WINTERSTEIN, A., KLEINMAN, L. C. & BOLEN, S. D. 2018. A transdisciplinary team approach to scoping reviews: the case of pediatric polypharmacy. *BMC Med Res Methodol,* 18**,** 102.

BALVANZ, P., DODGEN, L., QUINN, J., HOLLOWAY, T., HUDSPETH, S. & ENG, E. 2016. From Voice to Choice: African American Youth Examine Childhood Obesity in Rural North Carolina. *Prog Community Health Partnersh,* 10**,** 293-303.

BANYARD, V., EDWARDS, K., HERRINGTON, R., HOPFAUF, S., SIMON, B. & SHROLL, L. 2020. Using photovoice to understand and amplify youth voices to prevent sexual and relationship violence. *Journal of community psychology*.

BEARMAN, S. K., BAILIN, A., RODRIGUEZ, E. & BELLEVUE, A. 2020. Partnering with school providers to codesign mental health interventions: An open trial of Act & Adapt in urban public middle schools. *Psychology in the Schools,* 57**,** 1689-1709.

BEATRIZ, E. D., LINCOLN, A. K., ALDER, J., DALEY, N., SIMMONS, F., IBEH, K., FIGUEROA, C. & MOLNAR, B. E. 2018. Evaluation of a Teen Dating Violence Prevention Intervention among Urban Middle-School Youth Using Youth Participatory Action Research: Lessons Learned from Start Strong Boston. *J Fam Violence,* 33**,** 563-578.

BELL, S., AGGLETON, P., LOCKYER, A., FERGUSON, T., MURRAY, W., SILVER, B., KALDOR, J., MAHER, L. & WARD, J. 2021. Working with Aboriginal young people in sexual health research: a peer research methodology in remote Australia. *Qualitative Health Research,* 31**,** 16-28.

BENNINGER, E., SCHMIDT-SANE, M. & SPILSBURY, J. C. 2021. Conceptualizing Social Determinants of Neighborhood Health through a Youth Lens. *Child indicators research***,** 1-24.

BERGE, J. M., JIN, S. W., HANSON, C., DOTY, J., JAGARAJ, K., BRAATEN, K. & DOHERTY, W. J. 2016. Play it forward! A community-based participatory research approach to childhood obesity prevention. *Fam Syst Health,* 34**,** 15-30.

BIRNIE, K. A., DIB, K., OUELLETTE, C., DIB, M. A., NELSON, K., PAHTAYKEN, D., BAERG, K., CHORNEY, J., FORGERON, P., LAMONTAGNE, C., NOEL, M., POULIN, P. & STINSON, J. 2019. Partnering For Pain: a Priority Setting Partnership to identify patient-oriented research priorities for pediatric chronic pain in Canada. *CMAJ open,* 7**,** E654-E664.

BOATENG, M. A., AGYEI-BAFFOUR, E., ANGEL, S., ASARE, O., PREMPEH, B. & ENEMARK, U. 2021. Co-creation and prototyping of an intervention focusing on health literacy in management of malaria at community-level in Ghana. *Research involvement and engagement,* 7**,** 55.

BOOTE, J., JULIOUS, S., HORSPOOL, M., ELPHICK, H., SMITHSON, W. H. & NORMAN, P. 2016. PPI in the PLEASANT trial: involving children with asthma and their parents in designing an intervention for a randomised controlled trial based within primary care. *Prim Health Care Res Dev,* 17**,** 536-548.

BOYCHUCK, Z., ANDERSEN, J., BUSSIÈRES, A., FEHLINGS, D., KIRTON, A., LI, P., OSKOUI, M., RODRIGUEZ, C., SHEVELL, M., SNIDER, L., MAJNEMER, A. & GROUP, P. 2020. Use of consensus methods to determine the early clinical signs of cerebral palsy. *Paediatrics & Child Health (1205-7088),* 25**,** 300-307.

BRADY, L.-M., TEMPLETON, L., TONER, P., WATSON, J., EVANS, D., PERCY-SMITH, B. & COPELLO, A. 2018. Involving young people in drug and alcohol research. *Drugs and Alcohol Today,* 18**,** 28-38.

BRANDS, B., CHOMTHO, S., SUTHUTVORAVUT, U., CHIONG MENG BOEY, C., TANG, S. F., GODFREY, K. M., KOLETZKO, B. & FOR THE, E. S. P. G. 2020. Early Nutrition eAcademy Southeast Asia e-Learning for Enhancing Knowledge on Nutrition during the First 1000 Days of Life. *Nutrients,* 12.

BRESSAN, S., TITOMANLIO, L., GOMEZ, B., MINTEGI, S., GERVAIX, A., PARRI, N., DA DALT, L., MOLL, H. A., WAISMAN, Y., MACONOCHIE, I. K., OOSTENBRINK, R. & REPEM 2019.

Research priorities for European paediatric emergency medicine. *Archives of disease in childhood,* 104**,** 869-873.

BREWER, S. E., CRUMP, N. M. & O'LEARY, S. T. 2019. Patient-centered research priorities: A mixed-methods approach from the Colorado Children's Outcomes Network (COCONET). *Journal of the American Board of Family Medicine,* 32**,** 674-684.

BRITO, F. A., ZOELLNER, J. M., HILL, J., YOU, W., ALEXANDER, R., HOU, X. & ESTABROOKS, P. A. 2019. From Bright Bodies to iChoose: Using a CBPR Approach to Develop Childhood Obesity Intervention Materials for Rural Virginia. *SAGE open,* 9**,** 1-14.

BROCKIE, T. N., DANA-SACCO, G., LOPEZ, M. M. & WETSIT, L. 2017. Essentials of Research Engagement With Native American Tribes: Data Collection Reflections of a Tribal Research Team. *Prog Community Health Partnersh,* 11**,** 301-307.

BROWNE, S., BARRON, C., STAINES, A. & SWEENEY, M. R. 2020. Participatory Approaches to Understand Dietary Behaviours of Adolescents in the Secondary School Setting. *Nutrients,* 12**,** 3761.

CAMDEN, C., CAMPBELL, W., MISSIUNA, C., BERBARI, J., HÉGUY, L., GAUVIN, C., DOSTIE, R., IANNI, L., RIVARD, L. & ANABY, D. 2021. Implementing Partnering for Change in Québec: Occupational Therapy Activities and Stakeholders' Perceptions. *Canadian Journal of Occupational Therapy,* 88**,** 71-82.

CAMDEN, C., MEZIANE, S., MALTAIS, D., CANTIN, N., BROSSARD-RACINE, M., BERBARI, J. & COUTURE, M. 2019. Research and knowledge transfer priorities in developmental coordination disorder: Results from consultations with multiple stakeholders. *Health Expectations,* 22**,** 1156-1164.

CASALE, M., LANE, T., SELLO, L., KUO, C. & CLUVER, L. 2013. Conducting health survey research in a deep rural South African community: challenges and adaptive strategies. *Health Research Policy and Systems,* 11.

CENSE, M., GRAUW, S. D. & VERMEULEN, M. 2020. 'Sex Is Not Just about Ovaries.' Youth Participatory Research on Sexuality Education in The Netherlands. *International journal of environmental research and public health,* 17.

CHADWICK, J. Q., TULLIER, M. A., WOLBERT, L., COLEMAN, C., BRANAM, D. E., WHARTON, D. F., CANNADY, T. K., COPELAND, K. C. & SHORT, K. R. 2019. Collaborative implementation of a community-based exercise intervention with a partnering rural American Indian community. *Clinical Trials,* 16**,** 391-398.

CHATTERJEE, A., DAFTARY, G., CAMPBELL, M., GATISON, L., DAY, L., RAMSEY, K., GOLDMAN, R. & GILLMAN, M. W. 2016. “Can't we just have some Sazón?” student, family, and staff perspectives on a new school food program at a Boston high school. *Journal of School Health,* 86**,** 273-280.

CHENNEVILLE, T., GABBIDON, K., DRAKE, H. & RODRIGUEZ, L. 2019. Preliminary Findings From the HIV SEERs Project: A Community-Based Participatory Research Program to Reduce HIV Stigma Among Youth in Kenya. *JANAC: Journal of the Association of Nurses in AIDS Care,* 30**,** 462-473.

CHOPEL, A., SOTO, D., JOINER, B. J., BENITEZ, T., KONOFF, R., RIOS, L. & CASTELLANOS, E. 2019. Multilevel Factors Influencing Young Mothers' Breastfeeding: A Qualitative CBPR Study. *Journal of Human Lactation,* 35**,** 301-317.

CHOUDHRY, S., MCCLINTON-POWELL, L., SOLOMON, M., DAVIS, D., LIPTON, R., DARUKHANAVALA, A., STEENES, A., SELVARAJ, K., GIELISSEN, K. & LOVE, L. 2011. Power-up: a collaborative after-school program to prevent obesity in African American children. *Progress in community health partnerships: research, education, and action,* 5**,** 363.

CLARKE, S. 2021. An Exploration of the Child's Experience of Staying in Hospital from the Perspectives of Children and Children's Nurses using Child-Centered Methodology. *Comprehensive child and adolescent nursing***,** 1-14.

CLAUDIO, L., GILMORE, J., ROY, M. & BRENNER, B. 2018. Communicating environmental exposure results and health information in a community-based participatory research study. *BMC Public Health,* 18**,** 784.

CLEVERLEY, K., LENTERS, L. & MCCANN, E. 2020. "Objectively terrifying": a qualitative study of youth's experiences of transitions out of child and adolescent mental health services at age 18. *BMC psychiatry,* 20**,** 147.

COOKE, M., RICHARDS, J., TJONDRONEGORO, D., RAJ CHAKRABORTY, P., JAUNCEY-COOKE, J., ANDRESEN, E., THEODOROS, J., PATERSON, R., SCHULTS, J., RAITHATHA, B., WILSON, S. & ALCOCK, M. 2021. myPainPal: Co-creation of a mHealth app for the management of chronic pain in young people. *Informatics for Health & Social Care,* 46**,** 291-305.

CORDOVA, D., MUNOZ-VELAZQUEZ, J., MENDOZA LUA, F., FESSLER, K., WARNER, S., DELVA, J., ADELMAN, N., YOUTH LEADERSHIP, C., FERNANDEZ, A. & BAUERMEISTER, J. 2020. Pilot Study of a Multilevel Mobile Health App for Substance Use, Sexual Risk Behaviors, and Testing for Sexually Transmitted Infections and HIV Among Youth: Randomized Controlled Trial. *JMIR mHealth and uHealth,* 8**,** e16251.

CORRIGAN, P. W., SHAH, B. B., LARA, J. L., MITCHELL, K. T., COMBS-WAY, P., SIMMES, D. & JONES, K. L. 2018. Stakeholder perspectives on the stigma of fetal alcohol spectrum disorder. *Addiction Research & Theory,* 27**,** 170-177.

COY, K., BROCK, P., POMEROY, S., CADOGAN, J. & BECKETT, K. 2019. A Road Less Travelled: using Experience Based Co-Design to map children's and families' emotional journey following burn injury and identify service improvements. *Burns (03054179),* 45**,** 1848-1855.

CRAIG RUSHING, S. & STEPHENS, D. 2012. Tribal recommendations for designing culturally appropriate technology-based sexual health interventions targeting Native youth in the Pacific Northwest. *Am Indian Alsk Native Ment Health Res,* 19**,** 76-101.

CRISTIANI, V., KUMBAMU, A., ASIEDU, G. B., JOHNSON, S. K., GEWIRTZ O'BRIEN, J. R., ZIEBART, G., MOGEN, M. R., LYNCH, B. & KUMAR, S. 2021. Use of Community Based Participatory Research to Design Interventions for Healthy Lifestyle in an Alternative Learning Environment. *Journal of Primary Care & Community Health***,** 1-8.

CROSBY, S., LAIRD, K. & YOUNIE, S. 2020. Children and handwashing: Developing a resource to promote health and well-being in low and middle income countries. *Health Education Journal,* 79**,** 123-137.

CROSS, A., ROSENBAUM, P., GRAHOVAC, D., BROCKLEHURST, J., KAY, D., BAPTISTE, S. & GORTER, J. W. 2018. A Web-Based Knowledge Translation Resource for Families and Service Providers (The "F-Words" in Childhood Disability Knowledge Hub): Developmental and Pilot Evaluation Study. *JMIR Rehabil Assist Technol,* 5**,** e10439.

CROSS, A., ROSENBAUM, P., GRAHOVAC, D., KAY, D. & GORTER, J. W. 2015. Knowledge mobilization to spread awareness of the 'F-words' in childhood disability: lessons from a family-researcher partnership. *Child Care Health Dev,* 41**,** 947-53.

CRUDEN, G., FRERICHS, L., LICH, K. H., POWELL, B. J., LANIER, P. & BROWN, C. H. 2020. Developing a Multi-Criteria Decision Analysis Tool to Support the Adoption of Evidence-Based Child Maltreatment Prevention Programs. *Prevention science : the official journal of the Society for Prevention Research,* 21**,** 1059-1064.

CRUDGINGTON, H., COLLINGWOOD, A., LYLE, S., BRAY, L., MARTIN, R., GRINGRAS, P., PAL, D. K. & MORRIS, C. 2020. Mapping epilepsy-specific patient-reported outcome measures for children to a proposed core outcome set for childhood epilepsy. *Epilepsy and Behavior,* 112**,** 107372.

CUEVA, K., SCHMIDT, J. & CUEVA, M. 2021. Learning Together: Sharing Circles in Rural Alaska on Cancer Education Priorities for Youth. *Journal of cancer education : the official journal of the American Association for Cancer Education*.

CUNNINGHAM, R. M., CARTER, P. M., RANNEY, M. L., WALTON, M., ZEOLI, A. M., ALPERN, E. R., BRANAS, C., BEIDAS, R. S., EHRLICH, P. F., GOYAL, M. K., GOLDSTICK, J. E., HEMENWAY, D., HARGARTEN, S. W., KING, C. A., MASSEY, L., NGO, Q., PIZARRO, J., PROSSER, L., ROWHANI-RAHBAR, A. & RIVARA, F. 2019. Prevention of Firearm Injuries Among Children and Adolescents: Consensus-Driven Research Agenda from the Firearm Safety Among Children and Teens (FACTS) Consortium. *JAMA Pediatrics,* 173**,** 780-789.

CURRAN, J. A., GALLANT, A. J., ZEMEK, R., NEWTON, A. S., JABBOUR, M., CHORNEY, J., MURPHY, A., HARTLING, L., MACWILLIAMS, K., PLINT, A., MACPHEE, S., BISHOP, A. & CAMPBELL, S. G. 2019. Discharge communication practices in pediatric emergency care: a systematic review and narrative synthesis. *Syst Rev,* 8**,** 83.

DALY-SMITH, A., QUARMBY, T., ARCHBOLD, V. S. J., CORRIGAN, N., WILSON, D., RESALAND, G. K., BARTHOLOMEW, J. B., SINGH, A., TJOMSLAND, H. E., SHERAR, L. B., CHALKLEY, A., ROUTEN, A. C., SHICKLE, D., BINGHAM, D. D., BARBER, S. E., VAN SLUIJS, E., FAIRCLOUGH, S. J. & MCKENNA, J. 2020. Using a multi-stakeholder experience-based design process to co-develop the Creating Active Schools Framework. *International Journal of Behavioral Nutrition & Physical Activity,* 17**,** 1-12.

DANKER, J., STRNADOVÁ, I. & CUMMING, T. M. 2019. Picture my well-being: Listening to the voices of students with autism spectrum disorder. *Research in Developmental Disabilities,* 89**,** 130-140.

DAOUST, G., RUSHTON, P. W., RACINE, M., LEDUC, K., ASSILA, N. & DEMERS, L. 2021. Adapting the Wheelchair Skills Program for pediatric rehabilitation: recommendations from key stakeholders. *BMC Pediatrics,* 21**,** 1-13.

DAVIS, D. S., GOLDMON, M. V. & COKER-APPIAH, D. S. 2011. Using a community-based participatory research approach to develop a faith-based obesity intervention for African American children. *Health Promot Pract,* 12**,** 811-22.

DAVISON, K. K., JURKOWSKI, J. M., LI, K., KRANZ, S. & LAWSON, H. A. 2013. A childhood obesity intervention developed by families for families: results from a pilot study. *Int J Behav Nutr Phys Act,* 10.

DE HEER, B., HEFFERN, J. K., CHENEY, J. S., SECAKUKU, A. & BALDWIN, J. 2020. A Community-Based Evaluation of a Culturally Grounded, American Indian After-School Prevention Program: The Value of Practitioner-Researcher Collaboration. *American Indian and Alaska native mental health research (Online),* 27**,** 1-20.

DE ROSIS, S., PENNUCCI, F., NUTI, S. & NOTO, G. 2020. Healthy living and co-production: Evaluation of processes and outcomes of a health promotion initiative co-produced with adolescents. *International Journal of Environmental Research and Public Health,* 17**,** 1-19.

DEANS, E., RAVULO, J., BLIGNAULT, I. & CONROY, E. 2021. Understanding the needs of local youth to inform drug and alcohol prevention and harm reduction services: A qualitative study. *Health promotion journal of Australia : official journal of Australian Association of Health Promotion Professionals,* 32**,** 416-424.

DEAVENPORT-SAMAN, A., PIRIDZHANYAN, A., SOLOMON, O., PHILLIPS, Z., KUO, T. & YIN, L. 2019. Early childhood obesity among underserved families: A multilevel community-academic partnership. *American Journal of Public Health,* 109**,** 593-596.

DECAMP, L. R., ACOSTA, J., BOU DELGADO, L., GUERRERO VAZQUEZ, M. & POLK, S. 2021. Community partnerships in emerging immigrant communities: Lessons learned addressing Latino childhood weight disparities. *Public Health Nursing,* 38**,** 288-295.

DECAMP, L. R., ACOSTA, J., GUERRERO VAZQUEZ, M. & POLK, S. 2021. From Clinic to Community: Adapting Evidence-Based Weight Management for Overweight Latinx Children to Better Address Social Determinants. *Health promotion practice***,** 1524839921993054.

DELARA, E. W. 2019. Consequences of Childhood Bullying on Mental Health and Relationships for Young Adults. *Journal of Child & Family Studies,* 28**,** 2379-2389.

DENNEHY, R., CRONIN, M. & ARENSMAN, E. 2019. Involving young people in cyberbullying research: The implementation and evaluation of a rights-based approach. *Health expectations : an international journal of public participation in health care and health policy,* 22**,** 54-64.

DEWA, L. H., CRANDELL, C., CHOONG, E., JAQUES, J., BOTTLE, A., KILKENNY, C., LAWRENCE-JONES, A., DI SIMPLICIO, M., NICHOLLS, D. & AYLIN, P. 2021. CCopeY: A Mixed-Methods Coproduced Study on the Mental Health Status and Coping Strategies of Young People During COVID-19 UK Lockdown. *Journal of Adolescent Health,* 68**,** 666-675.

DEWA, L. H., LAVELLE, M., AYLIN, P., PICKLES, K., KALORKOTI, C., JAQUES, J. & PAPPA, S. 2019. Young adults' perceptions of using wearables, social media and other technologies to detect worsening mental health: A qualitative study. *PLoS ONE,* 14**,** e0222655.

DEWA, L. H., LAWRENCE-JONES, A., CRANDELL, C., JAQUES, J., PICKLES, K., LAVELLE, M., PAPPA, S. & AYLIN, P. 2020. Reflections, impact and recommendations of a co-produced qualitative study with young people who have experience of mental health difficulties. *Health Expectations: An International Journal of Public Participation in Health Care & Health Policy***,** No-Specified.

DIPIETRO, J. L., MACKIEWICZ SEGHETE, K. L., KRANS, E. E., SNIDER, K. E., BOWER, R., PARKER, K., GULLICKSON, J., POTTER, A. S., GARAVAN, H., VATALARO, T. C., THOMASON, M. E., SULLIVAN, E. L. & GRAHAM, A. M. 2021. Stakeholder Perspectives on Advancing Understanding of Prenatal Opioid Exposure and Brain Development From the iOPEN Consortium of the Healthy Brain and Child Development Study. *Frontiers in psychology,* 12**,** 698766.

DOGBA, M. J., DAHAN-OLIEL, N., SNIDER, L., GLORIEUX, F. H., DURIGOVA, M., PALOMO, T., CORDEY, M., BEDARD, M. H., BEDOS, C. & RAUCH, F. 2016. Involving Families with Osteogenesis Imperfecta in Health Service Research: Joint Development of the OI/ECE Questionnaire. *PLoS One,* 11**,** e0147654.

DOLWICK GRIEB, S. M., BERGSTEIN, R., GRIFFIN, B. & JENNINGS, J. M. 2019. Youth Voices on the Sexually Transmitted Infection Risk Environment: Community Violence, Chronic Trauma, and Sexual Health Outcomes. *Progress in community health partnerships : research, education, and action,* 13**,** 51-58.

DOMONEY, J., FULTON, E., STANLEY, N., MCINTYRE, A., HESLIN, M., BYFORD, S., BICK, D., RAMCHANDANI, P., MACMILLAN, H., HOWARD, L. M. & TREVILLION, K. 2019. For Baby's Sake: Intervention Development and Evaluation Design of a Whole-Family Perinatal Intervention to Break the Cycle of Domestic Abuse. *Journal of Family Violence,* 34**,** 539-551.

DONISI, V., GAJOFATTO, A., MAZZI, M. A., GOBBIN, F., BUSCH, I. M., GHELLERE, A., KLONOVA, A., RUDI, D., VITALI, F., SCHENA, F., DEL PICCOLO, L. & RIMONDINI, M. 2021. A Bio-

Psycho-Social Co-created Intervention for Young Adults With Multiple Sclerosis (ESPRIMO): Rationale and Study Protocol for a Feasibility Study. *Frontiers in psychology,* 12**,** 598726.

DOUBT, J., MEDLEY, S., CLUVER, L., WESSELS, I., ASNONG, C., MALUNGA, S., HODES, R., MAUCHLINE, K., VALE, B., RUPERT, C., GITTINGS, L., CHETTY, A. N., TOSKA, E., THABENG, M., ORKIN, K., DUNKLEY, Y., MEINCK, F., MYEKETSI, N., LASA, S., BOYES, M., PANTELIC, M., SHERR, L. & KUO, C. 2021. Power to participants: methodological and ethical reflections from a decade of adolescent advisory groups in South Africa. *AIDS Care - Psychological and Socio-Medical Aspects of AIDS/HIV,* 33**,** 858-866.

DRIEST, K., MACDONALD, D., FROST, E., SIVARAMAN, V., FURRU, R., GOLDSTEIN-LEEVER, A., LEMLE, S. & MULVIHILL, E. 2021. Targeted provider education and pre-visit planning increase rates of formal depression screening in childhood-onset SLE. *Pediatric Rheumatology,* 19**,** 116.

DUDOVITZ, R. N., RUSS, S., BERGHAUS, M., IRUKA, I. U., DIBARI, J., FONEY, D. M., KOGAN, M. & HALFON, N. 2021. COVID-19 and Children's Well-Being: A Rapid Research Agenda. *Maternal & Child Health Journal,* 25**,** 1655-1669.

DUFFY, E. W., LOTT, M. M., JOHNSON, E. J. & STORY, M. T. 2020. Developing a national research agenda to reduce consumption of sugar-sweetened beverages and increase safe water access and consumption among 0- to 5-year-olds: a mixed methods approach. *Public Health Nutrition,* 23**,** 22-33.

DURHAM, J., FA'AVALE, N., ZIESMAN, C., MALAMA, E., TAFA, S., TAITO, T., ETUALE, J., YARANAMUA, M., UTAI, U., SCHUBERT, L. & FA'AVALE, A. 2019. The impact and importance of place on health for young people of Pasifika descent in Queensland, Australia: A qualitative study towards developing meaningful health equity indicators. *International Journal for Equity in Health,* 18**,** 81.

DURL, J., DIETRICH, T., WILLIAMS, B. & RUNDLE-THIELE, S. 2021. Integrating student and teacher insights to a school-based alcohol program through co-design. *Health promotion international*.

DYKE, E., PENICAUD, S., HATCHARD, J., DAWSON, A.-M., MUNISHI, O. & JALAL, C. 2021. Girl-Powered Nutrition Program: Key Themes from a Formative Evaluation of a Nutrition Program Co-designed and Implemented by Adolescent Girls in Low- and Middle-Income Countries. *Current developments in nutrition,* 5**,** nzab083.

ELATTMA, A., LAVES, E., TABER, B., KARVONEN, K. L., HERRERA, M. C. & BAKKEN, E. H. 2020. Using Provider Incentives and an Opt-Out Strategy in a Successful Quality Initiative to Increase Chlamydia Screening. *Joint Commission Journal on Quality & Patient Safety,* 46**,** 326-334.

ENGLAND, K. J., EDWARDS, A. L., PAULSON, A. C., LIBBY, E. P., HARRELL, P. T. & MONDEJAR, K. A. 2021. Rethink Vape: Development and evaluation of a risk communication campaign to prevent youth E-cigarette use. *Addictive behaviors,* 113**,** 106664.

ENNALS, P., LESSING, K., SPIES, R., EGAN, R., HEMUS, P., DROPPERT, K., TIDHAR, M., WOOD, T., VAN DIJK, C., BRIDE, R., ASCHE, A., BENDALL, S. & SIMMONS, M. 2021. Co-producing

to understand what matters to young people living in youth residential rehabilitation services. *Early Intervention in Psychiatry*.

EVANS-AGNEW, R. A., POSTMA, J., CAMACHO, A. O., HERSHBERG, R. M., TRUJILIO, E. & TINAJERA, M. 2018. Development and Pilot Testing of a Bilingual Environmental Health Assessment Tool to Promote Asthma-friendly Childcares. *Prog Community Health Partnersh,* 12**,** 35-44.

FAKOYA, I., COLE, C., LARKIN, C., PUNTON, M., BROWN, E. & BALLONOFF SULEIMAN, A. 2021. Enhancing Human-Centered Design With Youth-Led Participatory Action Research Approaches for Adolescent Sexual and Reproductive Health Programming. *Health promotion practice***,** 15248399211003544.

FAREWELL, C. V., PUMA, J., MASON, M. A., PEIRCE, P., SHIMOMURA, M. & HARMS, M. 2020. Training Child Care Inspectors to Deliver Health Messaging: A Quality Improvement Pilot Project. *Health Promotion Practice,* 21**,** 188-197.

FARFAN, J. C., MARULANDA, S. C., ZAPATA, I. C. & CAINAS, N. E. 2018. Community Perspectives About Sociocultural Conditions Associated With Children's Health Among the Nasa People in Colombia. *Prog Community Health Partnersh,* 12**,** 279-288.

FARRANT, B. M., SHEPHERD, C. C. J., MICHIE, C., SCRINE, C., WRIGHT, M., ILICH, N., JONES, T. & PEARSON, G. 2019. Delivering Elder- and Community-Led Aboriginal Early Childhood Development Research: Lessons from the Ngulluk Koolunga Ngulluk Koort Project. *Children (Basel, Switzerland),* 6.

FASTRING, D., KEEL, K., COLBY, D., CONNER, J. M. & HILBERT, A. 2019. Head Start Centers Can Influence Healthy Behaviors: Evaluation of a Nutrition and Physical Activity Educational Intervention. *Journal of School Health,* 89**,** 698-704.

FEIN, E. H., WILLIAMS, P., CHUNG, P. J., MARTINEZ, A., STEWART, C., VALERI, A. & CHUNG, B. 2021. Engaging Community and Academic Partners to Explore Adolescent Emotional Well-Being. *Journal of Health Care for the Poor & Underserved,* 32**,** N.PAG-N.PAG.

FELDNER, H. A., LOGAN, S. W. & GALLOWAY, J. C. 2019. Mobility in pictures: a participatory photovoice narrative study exploring powered mobility provision for children and families. *Disability & Rehabilitation: Assistive Technology,* 14**,** 301-311.

FERDINANDS, A. R., OLSTAD, D. L., MILFORD, K. M., MAXIMOVA, K., NYKIFORUK, C. I. J. & RAINE, K. D. 2020. A Nutrition Report Card on food environments for children and youth: 5 years of experience from Canada. *Public Health Nutrition,* 23**,** 2088-2099.

FINE, M., TORRE, M. E., OSWALD, A. G. & AVORY, S. 2021. Critical participatory action research: Methods and praxis for intersectional knowledge production. *Journal of counseling psychology,* 68**,** 344-356.

FISHER, C. M., REECE, M., DODGE, B., WRIGHT, E., SHERWOOD-LAUGHLIN, C. & BALDWIN, K. 2010. Expanding our Reach: The Potential for Youth Development Professionals in Community-

Based Organizations to Provide Sexuality Information. *American Journal of Sexuality Education,* 5**,** 36-53.

FLEISCHHACKER, S., BYRD, R. R., RAMACHANDRAN, G., VU, M., RIES, A., BELL, R. A. & EVENSON, K. R. 2012. Tools for healthy tribes: improving access to healthy foods in Indian country. *Am J Prev Med,* 43**,** S123-9.

FLICKER, S., DANFORTH, J. Y., WILSON, C., OLIVER, V., LARKIN, J., RESTOULE, J.-P., MITCHELL, C., KONSMO, E., JACKSON, R. & PRENTICE, T. 2014. “Because we have really unique art”: Decolonizing Research with Indigenous Youth Using the Arts. *International Journal of Indigenous Health,* 10**,** 16-34.

FLICKER, S., TRAVERS, R., FLYNN, S., LARKIN, J., GUTA, A., SALEHI, R., POLE, J. D. & LAYNE, C. 2010. Sexual health research for and with urban youth: The Toronto Teen Survey story. *The Canadian Journal of Human Sexuality,* 19.

FLICKER, S., WILSON, C., MONCHALIN, R., OLIVER, V., PRENTICE, T., JACKSON, R., LARKIN, J., MITCHELL, C. & RESTOULE, J.-P. 2019. "Stay strong, stay sexy, stay native": Storying indigenous youth HIV prevention activism. *Action Research,* 17**,** 323-343.

FORD, T., RASMUS, S. & ALLEN, J. 2012. Being useful: achieving indigenous youth involvement in a community-based participatory research project in Alaska. *Int J Circumpolar Health,* 71**,** 1-7.

FOSTER, V. & YOUNG, A. 2013. Reflecting on participatory methodologies: research with parents of babies requiring neonatal care. *International Journal of Social Research Methodology,* 18**,** 91-104.

FRAUENBERGER, C., SPIEL, K. & MAKHAEVA, J. 2019. Thinking OutsideTheBox - Designing Smart Things with Autistic Children. *International journal of human-computer interaction,* 35**,** 666-678.

FUNK, A., VAN BOREK, N., TAYLOR, D., GREWAL, P., TZEMIS, D. & BUXTON, J. 2012. Climbing the "ladder of participation": engaging experiential youth in a participatory research project. *Can J Public Health,* 103**,** e288-92.

GAIHA, S. M., ZORRILLA, M., SACHNOFF, I., SMUIN, S., LAZARO, A., CEBALLOS, R. D., HALPERN-FELSHER, B. & RAZO, A. 2021. Development and Reach of the Stanford Tobacco Prevention Toolkit: Implementation of a Community-Based Participatory Approach. *The Journal of school health,* 91**,** 813-824.

GALVEZ, M., COLLINS, G., AMLER, R. W., DOZOR, A., KAPLAN-LISS, E., FORMAN, J., LARAQUE-ARENA, D., LAWRENCE, R., MILLER, R., MILLER, K., SHEFFIELD, P., ZAJAC, L. & LANDRIGAN, P. J. 2019. Building New York State Centers of Excellence in Children's Environmental Health: A Replicable Model in a Time of Uncertainty. *American journal of public health,* 109**,** 108-112.

GAMBLE, A., SAULTERS, M. M., CRANSTON, K. L., JONES, D. W., HERRING, S. J. & BEECH, B. M. 2020. Recruitment, Retention, and Engagement Strategies for Exercise Interventions With Rural

Antenatal Adolescents: Qualitative Interviews With WIC Providers. *Journal of Public Health Management & Practice,* 26**,** 497-502.

GARNETT, B., BEATTIE, H., KOLLER, S., MOORE, M., SCOTT, K., MASERONI, M. & HOLMES, B. 2019. Participatory Survey Data Analysis as Catalyst for Empowering Youth as School Health Change Agents. *Health Promotion Practice,* 20**,** 483-488.

GARVIN, T. M., WEISSENBURGER-MOSER BOYD, L., CHIAPPONE, A., BLASER, C., STORY, M., GERTEL-ROSENBERG, A., SHUELL, J., CHANG, D., WARD, D., PLUMLEE, C., BEETS, M. & YAROCH, A. L. 2019. Multisector Approach to Improve Healthy Eating and Physical Activity Policies and Practices in Early Care and Education Programs: The National Early Care and Education Learning Collaboratives Project, 2013-2017. *Preventing Chronic Disease,* 16**,** E94-E94.

GAUSMAN, J., LLOYD, D., KALLON, T., SUBRAMANIAN, S. V., LANGER, A. & AUSTIN, S. B. 2019. Clustered risk: An ecological understanding of sexual activity among adolescent boys and girls in two urban slums in Monrovia, Liberia. *Social science & medicine (1982),* 224**,** 106-115.

GEERLINKS, A. V., DIGOUT, C., BERNSTEIN, M., CHAN, A., MACPHEE, S., PAMBRUN, C., GALLANT, G., WYATT, L., FERNANDEZ, C. V. & PRICE, V. E. 2020. Improving Time to Antibiotics for Pediatric Oncology Patients With Fever and Suspected Neutropenia by Applying Lean Principles. *Pediatric Emergency Care,* 36**,** 509-514.

GEIGER, A., FENIGER-SCHAAL, R. & SHPIGELMAN, C.-N. 2020. The socio-emotional world of adolescents with intellectual disability: A drama therapy-based participatory action research. *Arts in Psychotherapy,* 70**,** 101679.

GELLATLY, J., BEE, P., KOLADE, A., HUNTER, D., GEGA, L., CALLENDER, C., HOPE, H. & ABEL, K. M. 2019. Developing an Intervention to Improve the Health Related Quality of Life in Children and Young People With Serious Parental Mental Illness. *Frontiers in psychiatry,* 10**,** 155.

GEPHART, S. M., HANSON, C., WETZEL, C. M., FLEINER, M., UMBERGER, E., MARTIN, L., RAO, S., AGRAWAL, A., MARIN, T., KIRMANI, K., QUINN, M., QUINN, J., DUDDING, K. M., CLAY, T., SAUBERAN, J., ESKENAZI, Y., PORTER, C., MSOWOYA, A. L., WYLES, C., AVENADO-RUIZ, M., VO, S., REBER, K. M. & DUCHON, J. 2017. NEC-zero recommendations from scoping review of evidence to prevent and foster timely recognition of necrotizing enterocolitis. *Matern Health Neonatol Perinatol,* 3**,** 23.

GERRITSEN, S., HARRE, S., RENKER-DARBY, A., SWINBURN, B., REES, D., BARTOS, A. E. & WATERLANDER, W. E. 2020. Community group model building as a method for engaging participants and mobilising action in public health. *International Journal of Environmental Research and Public Health,* 17**,** 3457.

GESSER-EDELSBURG, A., ORR, D., VERED-CHEN, L., ALAMOUR, Y., COHEN, R., SHAHBARI, N. A. E., HIJAZI, R. & SINGHAL, A. 2021. Creating safe spaces to prevent unintentional childhood injuries among the Bedouins in southern Israel: A hybrid model comprising positive deviance,

community-based participatory research, and entertainment-education. *PLoS ONE,* 16**,** e0257696.

GHANDOUR, E. K., LELAIDIER HOULD, L., FORTIER, F.-A., GÉLINAS, V., MELNICK, E. R., HESS, E. P., LANG, E. S., GRAVEL, J., PERRY, J. J., LE SAGE, N., TRUCHON, C., LEBLANC, A., DUBROVSKY, A. S., GAGNON, M.-P., OUELLET, M.-C., GAGNON, I., MCKENNA, S., LÉGARÉ, F., SAUVÉ, L. & VAN DE BELT, T. H. 2020. Adapting two American Decision Aids for Mild Traumatic Brain Injury to the Canadian Context Using the Nominal Group Technique. *Patient,* 13**,** 729-743.

GHIASVAND, H., ROSHANFEKR, P., ALI, D., ARDAKANI, H. M., STEPHENS, A. N. & ARMOON, B. 2020. Determinants of road traffic injuries in Iranian children; results from a National Representative Demographic- Health Survey 2010. *BMC Pediatrics,* 20**,** 1-7.

GILL, F. J., PIENAAR, C. & JONES, T. 2021. Using a 3 stage process to create a consumer research contact list in a paediatric health setting: the PARTICIPATE project. *Research involvement and engagement,* 7**,** 56.

GILLESPIE, J., MAGEE, E., STEWART, L. & WHITE, A. 2019. Eat, play, learn well-a novel approach to co-production and analysis grid for environments linked to obesity to engage local communities in a child healthy weight action plan. *Public Health,* 166**,** 99-107.

GILLISON, F., COONEY, G., WOOLHOUSE, V., DAVIES, A., DICKENS, F. & MARNO, P. 2017. Parents' perceptions of reasons for excess weight loss in obese children: a peer researcher approach. *Res Involv Engagem,* 3**,** 22.

GILLJAM, B.-M., ARVIDSSON, S., NYGREN, J. M. & SVEDBERG, P. 2020. Child participation in health care (ChiPaC)-Development and psychometric evaluation of a self-report instrument for children's participation in health care. *Journal of clinical nursing,* 29**,** 107-118.

GIORGI ROSSI, P., FERRARI, F., BONVICINI, L., AMARRI, S., FONTANA, M., LO SCOCCO, S., STREET, M. E., BASSI, A., GANUGI, G., MAESTRI, G., MORETTI, V., PRANDINI, R., DALL'AGLIO, L., FORACCHIA, M., DELLA GIUSTINA, C., FABBRI, A., FERRARI, A. M., FERRARI, E., PANZA, C., DAVOLI, A. M., GALLELLI, T., ILARI, B., PINOTTI, M., STORANI, S., VOLTA, A., TAMELLI, M., TROWBRIDGE, H. & VENTURELLI, F. 2020. Describing the Process and Tools Adopted to Cocreate a Smartphone App for Obesity Prevention in Childhood: Mixed Method Study. *JMIR mHealth and uHealth,* 8**,** e16165.

GLUBWILA, S., SRIPA, K. & THUMMAPHAN, P. 2021. The model of collaboration integration for preventing and solving the problem of youth violence in educational settings. *Current Psychology: A Journal for Diverse Perspectives on Diverse Psychological Issues***,** No-Specified.

GOFORTH, A. N., NICHOLS, L. M., SUN, J., VIOLANTE, A., CHRISTOPHER, K. & GRAHAM, N. 2021. Incorporating the indigenous evaluation framework for culturally responsive community engagement. *Psychology in the Schools***,** No-Specified.

GOLDENBERG, A. J., LLOYD-PURYEAR, M., BROSCO, J. P., THERRELL, B., BUSH, L., BERRY, S., BROWER, A., BONHOMME, N., BOWDISH, B., CHRYSLER, D., CLARKE, A., CRAWFORD, T., GOLDMAN, E., HINER, S., HOWELL, R. R., ORREN, D., WILFOND, B. S., WATSON, M., BIOETHICS & LEGAL WORKGROUP OF THE NEWBORN SCREENING TRANSLATIONAL RESEARCH, N. 2019. Including ELSI research questions in newborn screening pilot studies. *Genetics in medicine : official journal of the American College of Medical Genetics,* 21**,** 525-533.

GOLDENTHAL, H. J., RAVIV, T., BAKER, S., HOLLEY, C., SUMMERSETT WILLIAMS, F. & GOUZE, K. R. 2021. Development of a training and implementation model for school-based behavioral health interventions. *Psychology in the Schools***,** No-Specified.

GONZALEZ, M., DYCK HOLZINGER, S., SAXENA, S., CHOWDHURY, F., MARTENS, R., OSKOUI, M. & SHIKAKO-THOMAS, K. 2021. Informing the development of the Canadian Neurodiversity Platform (iCAN): What is important to parents of children with neurodevelopmental disabilities? *Child: care, health and development*.

GOODKIND, J. R., ROSS-TOLEDO, K., JOHN, S., LEE HALL, J., ROSS, L., FREELAND, L., COLLETA, E. & BECENTI-FUNDARK, T. 2011. Rebuilding TRUST: A Community, Multi-Agency, State, and University Partnership to Improve Behavioral Health Care for American Indian Youth, their Families, and Communities. *J Community Psychol,* 39**,** 452-477.

GOPALAN, G., BUNGER, A. C. & POWELL, B. J. 2020. Skills for Developing and Maintaining Community-Partnerships for Dissemination and Implementation Research in Children's Behavioral Health: Implications for Research Infrastructure and Training of Early Career Investigators. *Administration and policy in mental health,* 47**,** 227-243.

GOSS, H. R., MCDERMOTT, C., HICKEY, L., ISSARTEL, J., MEEGAN, S., MORRISSEY, J., MURRIN, C., PEERS, C., SMITH, C., SPILLANE, A. & BELTON, S. 2021. Understanding disadvantaged adolescents' perception of health literacy through a systematic development of peer vignettes. *BMC Public Health,* 21**,** 1-11.

GREER, A. E., MARTINEZ-CARRASCO, A., GOLDSMAN, D. & KNAUSENBERGER, A.-U. 2021. Walking Toward a Brighter Future: A Participatory Research Process to Advocate for Improved Walk-to-School Corridors. *Health Promotion Practice,* 22**,** 248-256.

GREGOROWSKI, A., BRENNAN, E., CHAPMAN, S., GIBSON, F., KHAIR, K., MAY, L. & LINDSAY-WATERS, A. 2013. An action research study to explore the nature of the nurse consultant role in the care of children and young people. *J Clin Nurs,* 22**,** 201-10.

GUERRERO, F., LUCAR, N., GARVICH CLAUX, M., CHIAPPE, M., PEREZ-LU, J., HINDIN, M. J., GONSALVES, L. & BAYER, A. M. 2020. Developing an SMS text message intervention on sexual and reproductive health with adolescents and youth in Peru. *Reproductive Health,* 17**,** 1-14.

GUINAUDIE, C., MIREAULT, C., TAN, J., PELLING, Y., JALALI, S., MALLA, A. & IYER, S. N. 2020. Shared Decision Making in a Youth Mental Health Service Design and Research Project: Insights From the Pan-Canadian ACCESS Open Minds Network. *Patient,* 13**,** 653-666.

HALL, C. L., BROWN, S., MARTIN, J. L., BROWN, N., WILLIAMS, L., GROOM, M. J., JAMES, M., SELBY, K., CLARKE, J., SAYAL, K. & HOLLIS, C. 2019. Consensus workshops on the development of an ADHD medication management protocol using QbTest: developing a clinical trial protocol with multidisciplinary stakeholders. *BMC medical research methodology,* 19**,** 126.

HALLETT, V., MUELLER, J., BREESE, L., HOLLETT, M., BERESFORD, B., IRVINE, A., PICKLES, A., SLONIMS, V., SCOTT, S., CHARMAN, T. & SIMONOFF, E. 2021. Introducing 'Predictive Parenting': A Feasibility Study of a New Group Parenting Intervention Targeting Emotional and Behavioral Difficulties in Children with Autism Spectrum Disorder. *Journal of Autism & Developmental Disorders,* 51**,** 323-333.

HAMDANI, S. U., HUMA, Z.-E., SULEMAN, N., WARRAITCH, A., MUZZAFAR, N., FARZEEN, M., MINHAS, F. A., RAHMAN, A. & WISSOW, L. S. 2021. Scaling-up school mental health services in low resource public schools of rural Pakistan: the Theory of Change (ToC) approach. *International journal of mental health systems,* 15**,** 8.

HANLEY, T., SEFI, A., GRAUBERG, J., PRESCOTT, J. & ETCHEBARNE, A. 2021. A Theory of Change for Web-Based Therapy and Support Services for Children and Young People: Collaborative Qualitative Exploration. *JMIR pediatrics and parenting,* 4**,** e23193.

HARLEY, K. G., KOGUT, K., MADRIGAL, D. S., CARDENAS, M., VERA, I. A., MEZA-ALFARO, G., SHE, J., GAVIN, Q., ZAHEDI, R., BRADMAN, A., ESKENAZI, B. & PARRA, K. L. 2016. Reducing Phthalate, Paraben, and Phenol Exposure from Personal Care Products in Adolescent Girls: Findings from the HERMOSA Intervention Study. *Environ Health Perspect,* 124**,** 1600-1607.

HARRIS, K. J., BROWN, B., PEDERSEN, M., SHANKLE, L., TRYON, M., PANARELLA, S. K. & SWANEY, G. 2019. Community Readiness Model for Prevention Planning: Addressing Childhood Obesity in American Indian Reservation Communities. *Journal of racial and ethnic health disparities,* 6**,** 1144-1156.

HART, K. M. & NEIL, N. 2020. Down syndrome caregivers' support needs: A mixed-method participatory approach. *Journal of Intellectual Disability Research***,** No-Specified.

HAVERINEN-SHAUGHNESSY, U., KHAN, S., BOULAFENTIS, J., GARCIA, C. & SHAUGHNESSY, R. 2020. Effects of educational efforts in tribal homes and schools to reduce asthma triggers, symptoms and missed school days. *International Journal of Hygiene & Environmental Health,* 228**,** N.PAG-N.PAG.

HELIMAKI, M., LAITILA, A. & KUMPULAINEN, K. 2021. "You helped me out of that darkness" Children as dialogical partners in the collaborative post-family therapy research interview. *Journal of marital and family therapy*.

HELM, S., LEE, W., HANAKAHI, V., GLEASON, K. & MCCARTHY, K. 2015. Using Photovoice with youth to develop a drug prevention program in a rural Hawaiian community. *American Indian and Alaska native mental health research (Online),* 22.

HENNESSY, M., BYRNE, M., LAWS, R., MC SHARRY, J., O'MALLEY, G. & HEARY, C. 2019. Childhood obesity prevention: priority areas for future research and barriers and facilitators to knowledge translation, coproduced using the nominal group technique. *Translational behavioral medicine,* 9**,** 759-767.

HERRMAN, J. W., GORDON, M., RAHMER, B., MOORE, C. C., HABERMANN, B. & HAIGH, K. M. 2017. Assessing the effectiveness of Wise Guys: A mixed-methods approach. *Am J Sex Educ,* 12**,** 395-408.

HILL, A. E., COPLEY, J. A., QUINLAN, T., NELSON, A., MCLAREN, C. F., WHITE, R., CASTAN, C. & BRODRICK, J. 2020. Real gains: development of a tool to measure outcomes for urban First Australian children accessing culturally responsive interprofessional therapy. *Journal of interprofessional care***,** 1-8.

HILL, J. L., ZOELLNER, J. M., BROCK, D. J., YOU, W., PRICE, B., ALEXANDER, R. C., FRISARD, M., BRITO, F., HOU, X. & ESTABROOKS, P. A. 2019. Participatory development and pilot testing of iChoose: an adaptation of an evidence-based paediatric weight management program for community implementation. *BMC public health,* 19**,** 122.

HODES, R., VALE, B., TOSKA, E., CLUVER, L., DOWSE, R. & ASHORN, M. 2019. 'Yummy or crummy?' The multisensory components of medicines-taking among HIV-positive youth. *Global Public Health,* 14**,** 284-299.

HOEEG, D., GRABOWSKI, D. & CHRISTENSEN, U. 2019. Co-designing an intervention to prevent overweight and obesity among young children and their families in a disadvantaged municipality: Methodological barriers and potentials. *International Journal of Environmental Research and Public Health,* 16**,** 5110.

HUH, J., CASTRO, Y., KIM, I., LEE, K. J., YIP, J., ROLDAN, W., KSHIRSAGAR, S., RASTOGI, P., MILLER, K. A. & COCKBURN, M. 2021. Making of Mobile SunSmart: Co-designing a Just-in-Time Sun Protection Intervention for Children and Parents. *International journal of behavioral medicine*.

HUNT, M., PONNUSAMY, R., GOULET, A., ANTHONYPILLAI, C., MUTHUKARUPPAN, S. S., BHARATHWAJ, A., THOMAS, A., ARCHAMBAULT, P. S., GARNETT, C., STORR, C. & KRISHNA, D. 2020. An integrated knowledge translation project to develop, implement, and evaluate a train-the-trainer program at a community rehabilitation program in Tamil Nadu, India. *Disability and rehabilitation***,** 1-10.

HURTUBISE, K., BROUSSELLE, A. & CAMDEN, C. 2020. Using collaborative logic analysis evaluation to test the program theory of an intensive interdisciplinary pain treatment for youth with pain-related disability. *Paediatric and Neonatal Pain,* 2**,** 113-130.

HURTUBISE, K., BROUSSELLE, A., NOEL, M. & CAMDEN, C. 2020. What really matters in pediatric chronic pain rehabilitation? Results of a multi-stakeholder nominal group technique study. *Disability & Rehabilitation,* 42**,** 1675-1686.

HURTUBISE, K., PRATTE, G., HAMEL, C., CLAPPERTON, I. & CAMDEN, C. 2021. Rethinking early intervention rehabilitation services for children with motor difficulties: engaging stakeholders in the conceptualization of telerehabilitation primary care. *Disability and rehabilitation*, 1-10.

JABAREEN, R. & ZLOTNICK, C. 2021. The Cultural and Methodological Factors Challenging the Success of the Community-Based Participatory Research Approach When Designing a Study on Adolescents Sexuality in Traditional Society. *Qualitative health research,* 31**,** 887-897.

JACKSON, S. M., GESUALDO, F., TOZZI, A. E., DAVERIO, M. & PEREZ, S. L. 2020. Improving Informed Consent for Novel Vaccine Research in a Pediatric Hospital Setting Using a Blended Research-Design Approach. *Frontiers in Pediatrics,* 8**,** 520803.

JARDINE, C. G. & JAMES, A. 2012. Youth researching youth: benefits, limitations and ethical considerations within a participatory research process. *Int J Circumpolar Health,* 71**,** 1-9.

JENKINS, A. M., BURNS, D., HORICK, R., SPICER, B., VAUGHN, L. M. & WOODWARD, J. 2021. Adolescents and Young Adults With Spina Bifida Transitioning to Adulthood: A Comprehensive Community-Based Needs Assessment. *Academic Pediatrics,* 21**,** 858-867.

JENKINS, E. K., BUNGAY, V., PATTERSON, A., SAEWYC, E. M. & JOHNSON, J. L. 2018. Assessing the impacts and outcomes of youth driven mental health promotion: A mixed-methods assessment of the Social Networking Action for Resilience study. *J Adolesc,* 67**,** 1-11.

JULIEN, C., BRAY, D., LEE, S., CASTELLI, D., BURSON, S. & JUNG, Y. 2021. Project SMART: A cooperative educational game to increase physical activity in elementary schools. *Smart Health,* 19**,** 100163.

JUNG, Y., BURSON, S. L., JULIEN, C., BRAY, D. F. & CASTELLI, D. M. 2021. Development of a School-Based Physical Activity Intervention Using an Integrated Approach: Project SMART. *Frontiers in psychology,* 12**,** 648625.

JURKOWSKI, J. M., GREEN MILLS, L. L., LAWSON, H. A., BOVENZI, M. C., QUARTIMON, R. & DAVISON, K. K. 2013. Engaging low-income parents in childhood obesity prevention from start to finish: a case study. *J Community Health,* 38**,** 1-11.

KACZYNSKI, K., ELY, E., GORDON, D., VINCENT, C., WADDELL, K., WITTMAYER, K. & BERNHOFER, E. 2019. The Pediatric American Pain Society Patient Outcomes Questionnaire (Pediatric APS-POQ): Development and Initial Psychometric Evaluation of a Brief and Comprehensive Measure of Pain and Pain Outcomes in Hospitalized Youth. *Journal of Pain,* 20**,** N.PAG-N.PAG.

KAKAR, R., COMBS, R., ALI, N., MUVUKA, B. & PORTER, J. 2020. Enhancing the design and utilization of asthma action plans through community-based participatory research in an urban african american community. *Patient Education and Counseling***,** No-Specified.

KANG, Y., CHO, M., CHO, Y., RAHMAN, M. M., DUTTA, M. L. & HAN, S. 2021. Design of a collaborative monitoring and evaluation system for a community-based nutrition project in rural Bangladesh. *Evaluation and program planning,* 84**,** 101892.

KATZ, J. R., MARTINEZ, T. & PAUL, R. 2011. Community-based participatory research and American Indian/Alaska Native nurse practitioners: a partnership to promote adolescent health. *J Am Acad Nurse Pract,* 23**,** 298-304.

KATZ-WISE, S. L., PULLEN SANSFACON, A., BOGART, L. M., ROSAL, M. C., EHRENSAFT, D., GOLDMAN, R. E. & BRYN AUSTIN, S. 2019. Lessons from a community-based participatory research study with transgender and gender nonconforming youth and their families. *Action Research,* 17**,** 186-207.

KESTER, K. R. & LUCYSHYN, J. M. 2019. Co-creating a school-based Facing Your Fears anxiety treatment for children with autism spectrum disorder: A model for school psychology. *Psychology in the Schools,* 56**,** 824-839.

KHAYYAT KHOLGHI, M., BARTLETT, G., PHILLIPS, M., SALSBERG, J., MCCOMBER, A. M. & MACAULAY, A. C. 2018. Evaluating an Indigenous health curriculum for diabetes prevention: engaging the community through talking circles and knowledge translation of results. *Fam Pract,* 35**,** 80-87.

KIM, H., SHON, S. & SHIN, H. 2020. Exploring the unmet needs for creating an enabling environment for nurturing care to promote migrant child health in Bishkek, Kyrgyzstan: A theory-guided community-based participatory action research. *Evaluation and Program Planning,* 80.

KING, R. J., HEISEY-GROVE, D. M., GARRETT, N., SCOTT, K. A., DALEY, M. F., HAEMER, M. A., PODILA, P., BLOCK, J. P., CARTON, T., GREGOROWICZ, A. J., MORK, K. P., PORTER, R. M., CHUDNOV, D. L., JELLISON, J., KRAUS, E. M., HARRISON, M. R., SUCOSKY, M. S., ARMSTRONG, S. & GOODMAN, A. B. 2021. The Childhood Obesity Data Initiative: A Case Study in Implementing Clinical-Community Infrastructure Enhancements to Support Health Services Research and Public Health. *Journal of public health management and practice : JPHMP*.

KNIGHT, C. C., SELLECK, C. S., WAKEFIELD, R., HORTON, J. E., WILSON, M. E. & HARPER, D. C. 2020. Development of an academic practice partnership to improve maternal child health. *Journal of Professional Nursing,* 36**,** 116-122.

KOLLER, D. & MCLAREN, C. 2014. Children's Emotional Responses to a Paediatric Hospital Atrium. *Children & Society,* 28**,** 451-464.

KRAMER, J. M. & SCHWARTZ, A. E. 2018. Development of the Pediatric Disability Inventory-Patient Reported Outcome (PEDI-PRO) measurement conceptual framework and item candidates. *Scand J Occup Ther,* 25**,** 335-346.

KULBOK, P. A., MESZAROS, P. S., BOND, D. C., THATCHER, E., PARK, E., KIMBRELL, M. & SMITH-GREGORY, T. 2015. Youths as partners in a community participatory project for substance use prevention. *Fam Community Health,* 38**,** 3-11.

KURU, K., ANSELL, D., JONES, M., WATKINSON, B. J., CASWELL, N., LEATHER, P., LANCASTER, A., SUGDEN, P., BRIGGS, E., DAVIES, C., OH, T. C., BENNETT, K. & DE GOEDE, C. 2020.

Intelligent autonomous treatment of bedwetting using non-invasive wearable advanced mechatronics systems and MEMS sensors : Intelligent autonomous bladder monitoring to treat NE. *Medical & Biological Engineering & Computing,* 58**,** 943-965.

LAKHANPAUL, M., CULLEY, L., ROBERTSON, N., ALEXANDER, E. C., BIRD, D., HUDSON, N., JOHAL, N., MCFEETERS, M., HAMLYN-WILLIAMS, C., MANIKAM, L., BOO, Y. Y., LAKHANPAUL, M. & JOHNSON, M. R. D. 2020. A structured collaborative approach to intervention design using a modified intervention mapping approach: a case study using the Management and Interventions for Asthma (MIA) project for South Asian children. *BMC Medical Research Methodology,* 20**,** 1-16.

LANE, H. G., PORTER, K. J., HECHT, E., HARRIS, P. & ZOELLNER, J. M. 2019. A Participatory Process to Engage Appalachian Youth in Reducing Sugar-Sweetened Beverage Consumption. *Health promotion practice,* 20**,** 258-268.

LANGDON, S. E., GOLDEN, S. L., ARNOLD, E. M., MAYNOR, R. F., BRYANT, A., FREEMAN, V. K. & BELL, R. A. 2016. Lessons Learned From a Community-Based Participatory Research Mental Health Promotion Program for American Indian Youth. *Health Promot Pract,* 17**,** 457-63.

LARSON, K. L., BALLARD, S. M., ELLIS, D. F., PEERY, J. G., CARY, J. E., LEVY, R. L., NELSON, T. B. & SCROGGS, L. B. 2020. A Contextual Approach to Inform a Mobile Health Application for Adolescent Health. *Journal of Child & Family Studies,* 29**,** 3420-3432.

LAS HAYAS, C., IZCO-BASURKO, I., FULLAONDO, A., DE MANUEL KEENOY, E., GABRIELLI, S., DONISI, V., CARBONE, S., RIZZI, S., ZWIEFKA, A., HJEMDAL, O., MOROTE, R., ANYAN, F., GUDMUNDSDOTTIR, D. G., KNOOP, H. H., LEDERTOUG, M. M., TANGE, N., OLAFSDOTTIR, A. S., KALDALONS, I., JONSDOTTIR, B. J., KROLICKA-DEREGOWSKA, A., MAZUR, I., GONZALEZ-PINTO, A., VERGARA, I., GONZALEZ, N. & MAR MEDINA, J. 2019. UPRIGHT, a resilience-based intervention to promote mental well-being in schools: study rationale and methodology for a European randomized controlled trial. *BMC public health,* 19**,** 1413.

LEE, J. P., LIPPERMAN-KREDA, S., SAEPHAN, S. & KIRKPATRICK, S. 2013. Tobacco environment for Southeast Asian American youth: results from a participatory research project. *J Ethn Subst Abuse,* 12**,** 30-50.

LEE, J. P., PAGANO, A., KIRKPATRICK, S., LE, N., ERCIA, A. & LIPPERMAN-KREDA, S. 2019. Using photovoice to develop critical awareness of tobacco environments for marginalized youth in California. *Action Research,* 17**,** 510-527.

LEE, R. E., SOLTERO, E. G., LEDOUX, T. A., SAHNOUNE, I., SAAVADRA, F., MAMA, S. K. & MCNEILL, L. H. 2019. Sustainability via Active Garden Education: Translating Policy to Practice in Early Care and Education. *Journal of School Health,* 89**,** 257-266.

LEFF, S. S., THOMAS, D. E., VAUGHN, N. A., THOMAS, N. A., MACEVOY, J. P., FREEDMAN, M. A., ABDUL-KABIR, S., WOODLOCK, J., GUERRA, T., BRADSHAW, A. S., WOODBURN, E. M., MYERS, R. K. & FEIN, J. A. 2010. Using community-based participatory research to develop

the PARTNERS youth violence prevention program. *Prog Community Health Partnersh,* 4**,** 207-16.

LEHTONEN, K., VANSKA, N., SIPARI, S., JEGLINSKY, I. & KINNUNEN, A. 2021. Co-development of the CMAP Book: a tool to enhance children's participation in pediatric rehabilitation. *Disability and rehabilitation***,** 1-11.

LEVY, I. P. & ADJAPONG, E. S. 2020. Toward culturally competent school counseling environments: Hip-hop studio construction. *The Professional Counselor,* 10**,** 266-284.

LIGHTFOOT, A. F., THATCHER, K., SIMAN, F. M., ENG, E., MERINO, Y., THOMAS, T., COYNE-BEASLEY, T. & CHAPMAN, M. V. 2019. "What I wish my doctor knew about my life": Using photovoice with immigrant Latino adolescents to explore barriers to healthcare. *Qualitative social work : QSW : research and practice,* 18**,** 60-80.

LIGHTFOOT, A. F., WOODS, B. A., JACKSON, M., RIGGINS, L., KRIEGER, K., BRODIE, K., GRAY, P. & HOWARD, D. L. 2012. "In my house": laying the foundation for youth HIV prevention in the Black church. *Prog Community Health Partnersh,* 6**,** 451-6.

LINDQUIST-GRANTZ, R. & ABRACZINSKAS, M. 2020. Using Youth Participatory Action Research as a Health Intervention in Community Settings. *Health Promotion Practice,* 21**,** 573-581.

LIU, F. F., LEW, A., ANDES, E., MCNAMARA, S., CASSIDY, J., WHITMORE, S., PLUNKETT, R. & ONG, T. 2020. Implementation strategies for depression and anxiety screening in a pediatric cystic fibrosis center: A quality improvement project. *Pediatric Pulmonology,* 55**,** 3328-3336.

LOESCHER, L. J., RAWDIN, S., MACHAIN, T., EMRICK, G., PASVOGEL, A., SPARTONOS, D., JOHNSON, R. E. & CAMPAS, D. 2019. Implementation of Project Students Are Sun Safe (SASS) in Rural High Schools Along the Arizona-Mexico Border. *Journal of Cancer Education,* 34**,** 259-268.

LOGAN, D. E., SIMONS, L. E., CARUSO, T. J., GOLD, J. I., GREENLEAF, W., GRIFFIN, A., KING, C. D., MENENDEZ, M., OLBRECHT, V. A., RODRIGUEZ, S., SILVIA, M., STINSON, J. N., WANG, E., WILLIAMS, S. E., WILSON, L., LOGAN, D. E., SIMONS, L. E., CARUSO, T., KING, C. & OLBRECHT, V. 2021. Leveraging Virtual Reality and Augmented Reality to Combat Chronic Pain in Youth: Position Paper From the Interdisciplinary Network on Virtual and Augmented Technologies for Pain Management. *Journal of Medical Internet Research,* 23**,** N.PAG-N.PAG.

LONGO, C., RAHIMZADEH, V. & BARTLETT, G. 2021. Communication of Pharmacogenomic test results and treatment plans in pediatric oncology: deliberative stakeholder consultations with parents. *BMC Palliative Care,* 20**,** 1-12.

LUND, J., RADFORD, A., KUSHNER, L., TOOMBS, E., BOBINSKI, T., DIXON, J., DREBIT, M., BYZEWSKI, J. & MUSHQUASH, C. J. 2021. Client and staff experiences assessing adverse childhood experiences in a clinical setting: Results from the First Nations ACE Study. *Child Abuse and Neglect,* 121**,** 105263.

MACARTHUR, C., WALSH, C. M., BUCHANAN, F., KAROLY, A., PIRES, L., MCCREATH, G. & JONES, N. L. 2021. Development of the patient-oriented research curriculum in child health (PORCCH). *Research involvement and engagement,* 7**,** 27.

MACRIDIS, S., GARCIA BENGOECHEA, E., MCCOMBER, A. M., JACOBS, J., MACAULAY, A. C. & MEMBERS OF THE KAHNAWAKE SCHOOLS DIABETES PREVENTION PROJECT-SCHOOL TRAVEL PLANNING, C. 2016. Active transportation to support diabetes prevention: Expanding school health promotion programming in an Indigenous community. *Eval Program Plann,* 56**,** 99-108.

MAGI, K., KALMA, M., MAESTU, E., KULL, M., MOOSES, K., VIHALEMM, T., UIBU, M. & KORP, L. 2021. Developing a comprehensive school-based physical activity program with flexible design - from pilot to national program. *BMC public health,* 21**,** 92.

MAITLAND, N., WARDLE, K., WHELAN, J., JALALUDIN, B., CREIGHTON, D., JOHNSTONE, M., HAYWARD, J. & ALLENDER, S. 2021. Tracking implementation within a community-led whole of system approach to address childhood overweight and obesity in south west Sydney, Australia. *BMC Public Health,* 21**,** 1-11.

MANCE, G. A., RODGERS, C. R. R., ROBERTS, D. & TERRY, A. 2020. Deeply rooted: Maximizing the strengths of a Historically Black University and community-based participatory research to understand environmental stressors and camong Black youth. *American Journal of Community Psychology,* 66**,** 256-266.

MARSH, V. M., KAMUYA, D. M., MLAMBA, A. M., WILLIAMS, T. N. & MOLYNEUX, S. S. 2010. Experiences with community engagement and informed consent in a genetic cohort study of severe childhood diseases in Kenya. *BMC Medical Ethics,* 11**,** 13.

MARTEL, S., HEIDEBRECHT, C., D'SILVA, C., SINGH, N., FIERHELLER, D. & ZENLEA, I. 2021. Building a community-based participatory approach to child, youth, and family health: Learnings from organizational engagement in the Peel Region of Ontario. *Families, systems & health : the journal of collaborative family healthcare*.

MASTERSON, E. E., YOUNGLOVE, L. B., KRENZ, J. E., TCHONG FRENCH, M. I., RIEDERER, A. M., MIN, E., JANSEN, K. L., BABADI, R. S., PEREZ, A., TORRES, E., SAMPSON, P. D., METWALI, N., THORNE, P. S., AISENBERG, G., FARQUHAR, S. A. & KARR, C. J. 2020. The home air in agriculture pediatric intervention (HAPI) trial: Rationale and methods. *Contemporary Clinical Trials,* 96**,** 106085.

MATHEWS, J. R., MATHEWS, T. L. & MWAJA, E. 2010. "Girls take charge": a community-based participatory research program for adolescent girls. *Prog Community Health Partnersh,* 4**,** 17-24.

MBACHU, C. O., AGU, I. C., ONWUJEKWE, O. & CLARA AGU, I. 2020. Collaborating to co-produce strategies for delivering adolescent sexual and reproductive health interventions: processes and experiences from an implementation research project in Nigeria. *Health Policy & Planning,* 35**,** ii84-ii97.

MCCALMAN, J., TSEY, K., BAINBRIDGE, R., SHAKESHAFT, A., SINGLETON, M. & DORAN, C. 2013. Tailoring a response to youth binge drinking in an Aboriginal Australian community: a grounded theory study. *BMC Public Health,* 13**,** 726.

MCCREARY, M., AREVIAN, A. C., BRADY, M., MOSQUEDA CHICHITS, A. E., ZHANG, L., TANG, L. & ZIMA, B. 2019. A Clinical Care Monitoring and Data Collection Tool (H3 Tracker) to Assess Uptake and Engagement in Mental Health Care Services in a Community-Based Pediatric Integrated Care Model: Longitudinal Cohort Study. *JMIR mental health,* 6**,** e12358.

MCDONALD, C. C., RICHMOND, T. S., GUERRA, T., THOMAS, N. A., WALKER, A., BRANAS, C. C., TENHAVE, T. R., VAUGHN, N. A., LEFF, S. S. & HAUSMAN, A. J. 2012. Methods for linking community views to measureable outcomes in a youth violence prevention program. *Prog Community Health Partnersh,* 6**,** 499-506.

MCERLANE, F., ARMITT, G., COBB, J., BAILEY, K., CLEARY, G., DOUGLAS, S., LUNT, L., RASHID, A., SAMPATH, S., SHOOP-WORRALL, S., SMITH, N., FOSTER, H. & THOMSON, W. 2020. CAPTURE-JIA: a consensus-derived core dataset to improve clinical care for children and young people with juvenile idiopathic arthritis. *Rheumatology,* 59**,** 137-145.

MCKAY, M. M., SENSOY BAHAR, O. & SSEWAMALA, F. M. 2020. Implementation science in global health settings: Collaborating with governmental & community partners in Uganda. *Psychiatry Research,* 283**,** 112585.

MCLOUGHLIN, G. M., VAZOU, S., LIECHTY, L., TORBERT, A., LANNINGHAM-FOSTER, L., ROSENKRANZ, R. R. & WELK, G. J. 2021. Transdisciplinary Approaches for the Dissemination of the SWITCH School Wellness Initiative Through a Distributed 4-H/Extension Network. *Child & Youth Care Forum,* 50**,** 99-120.

MERRITT, R. K., DE GROOT, J., ALMAJALI, L. & PATEL, N. 2021. Using Community-Based Prevention Marketing to Generate Demand for Healthy Diets in Jordan. *Nutrients,* 13**,** 3068-3068.

MILTON, A. C., STEWART, E., OSPINA-PINILLOS, L., DAVENPORT, T. & HICKIE, I. B. 2021. Participatory Design of an Activities-Based Collective Mentoring Program in After-School Care Settings: Connect, Promote, and Protect Program. *JMIR pediatrics and parenting,* 4**,** e22822.

MIXER, S. J., CARSON, E., MCARTHUR, P. M., ABRAHAM, C., SILVA, K., DAVIDSON, R., SHARP, D. & CHADWICK, J. 2015. Nurses in Action: A Response to Cultural Care Challenges in a Pediatric Acute Care Setting. *J Pediatr Nurs,* 30**,** 896-907.

MONTREUIL, M., MCHARG, L., CARNEVALE, F. A. & THIBEAULT, C. 2020. Moral Experiences of Crisis Management in a Child Mental Health Setting: A Participatory Hermeneutic Ethnographic Study. *Culture, medicine and psychiatry,* 44**,** 80-109.

MORALES-ALEMÁN, M. M., FERRETI, G. & SCARINCI, I. C. 2020. "I Don't Like Being Stereotyped, I Decided I Was Never Going Back to the Doctor": Sexual Healthcare Access Among Young Latina Women in Alabama. *Journal of Immigrant & Minority Health,* 22**,** 645-652.

MORGAN, J., PHILLIPS, B. & DEPANI, S. 2019. What do families want to improve in the management of paediatric febrile neutropenia during anti-cancer treatment? Report of a patient/public involvement group. *BMJ Paediatrics Open,* 3**,** e000398.

MORGAN, K., VAN GODWIN, J., DARWENT, K. & FILDES, A. 2019. Formative research to develop a school-based, community-linked physical activity role model programme for girls: CHoosing Active Role Models to INspire Girls (CHARMING). *BMC Public Health,* 19**,** N.PAG-N.PAG.

MORRIS, S. L., HOSPITAL, M. M., WAGNER, E. F., LOWE, J., THOMPSON, M. G., CLARKE, R. & RIGGS, C. 2021. SACRED Connections: A University-Tribal Clinical Research Partnership for School-Based Screening and Brief Intervention for Substance Use Problems among Native American Youth. *Journal of ethnic & cultural diversity in social work,* 30**,** 149-162.

MUGO, C., NJUGUNA, I., NDUATI, M., OMONDI, V., OTIENO, V., NYAPARA, F., MABELE, E., MORAA, H., SHERR, K., INWANI, I., MALECHE-OBIMBO, E., WAMALWA, D., JOHN-STEWART, G., SLYKER, J. & WAGNER, A. D. 2020. From research to international scale-up: stakeholder engagement essential in successful design, evaluation and implementation of paediatric HIV testing intervention. *Health policy and planning,* 35**,** 1180-1187.

MUTISYA, M., MARKEY, O., ROUSHAM, E. K., GRIFFITHS, P. L., HAYCRAFT, E., CHINTSANYA, J. M. N., PRADEILLES, R., KIMANI-MURAGE, E. W., MADISE, N. J., MUNTHALI, A. C., KALIMBIRA, A. & HOLDSWORTH, M. 2021. Improving nutritional status among urban poor children in sub-Saharan Africa: An evidence-informed Delphi-based consultation. *Maternal and Child Nutrition,* 17**,** e13099.

NECHELES, J. W., CHUNG, E. Q., HAWES-DAWSON, J., RYAN, G. W., WILLIAMS, S. B., HOLMES, H. N., WELLS, K. B., VAIANA, M. E. & SCHUSTER, M. A. 2007. The Teen Photovoice Project: a pilot study to promote health through advocacy. *Prog Community Health Partnersh,* 1**,** 221-9.

NOONE, J., SULLIVAN, M., MCKINNIS, N. C., ALLEN, T. L., REGALADO, C. & ESQUEDA, T. 2016. Latino youth participation in community-based participatory research to reduce teen pregnancy disparities. *Children and Youth Services Review,* 63**,** 36-39.

NYAKATO, V. N., ACHEN, C., CHAMBERS, D., KAZIGA, R., OGUNNAYA, Z., WRIGHT, M. & KOOLS, S. 2021. Very young adolescent perceptions of growing up in rural southwest Uganda: Influences on sexual development and behavior. *African Journal of Reproductive Health,* 25**,** 50-64.

O'CONNOR, E. C., HUTAIN, J., CHRISTENSEN, M., KAMARA, M. S., CONTEH, A., SARRIOT, E., SAMBA, T. T. & PERRY, H. B. 2019. Piloting a participatory, community-based health information system for strengthening community-based health services: findings of a cluster-randomized controlled trial in the slums of Freetown, Sierra Leone. *Journal of Global Health,* 9**,** 1-15.

OBEROI, A. R., TOWRY, L., EILENBERG, J. S., LUN, P., LERRO, G., ALDERFER, M. A. & LONG, K. A. 2019. Improving support to siblings of children with cancer through a community-academic partnership. *Clinical Practice in Pediatric Psychology,* 7**,** 371-382.

OCHIENG, L., AMAUGO, L. & OCHIENG, B. M. N. 2021. Developing healthy weight maintenance through co-creation: a partnership with Black African migrant community in East Midlands. *European Journal of Public Health,* 31**,** 487-493.

OGENCHUK, M., GRAHAM, H., ST PIERRE, R., USWAK, G., WEILER, R. & RAMSDEN, V. R. 2021. Community led: celebrating wellness with a smile. *International journal of circumpolar health,* 80**,** 1962023.

OLLIS, D., COLL, L. & HARRISON, L. 2019. Negotiating sexuality education with young people: Ethical pitfalls and provocations. *American Journal of Sexuality Education,* 14**,** 186-202.

OTT, M. A., CAMPBELL, J., IMBURGIA, T. M., YANG, Z., TU, W. & AUERSWALD, C. L. 2018. Community Engagement and Venue-Based Sampling in Adolescent Male Sexually Transmitted Infection Prevention Research. *J Adolesc Health,* 62**,** S58-S64.

PARTRIDGE, S. R., RAESIDE, R., LATHAM, Z., SINGLETON, A. C., HYUN, K., GRUNSEIT, A., STEINBECK, K. & REDFERN, J. 2019. 'Not to Be Harsh but Try Less to Relate to 'the Teens' and You'll Relate to Them More': Co-Designing Obesity Prevention Text Messages with Adolescents. *International journal of environmental research and public health,* 16.

PAVLOPOULOU, G. & DIMITRIOU, D. 2020. In their own words, in their own photos: Adolescent females' siblinghood experiences, needs and perspectives growing up with a preverbal autistic brother or sister. *Research in Developmental Disabilities,* 97**,** N.PAG-N.PAG.

PEREA, F. C., SAYLES, N. R., REICH, A. J., KOOMAS, A., MCMANN, H. & SPRAGUE MARTINEZ, L. S. 2019. "Mejorando Nuestras Oportunidades": Engaging Urban Youth in Environmental Health Assessment and Advocacy to Improve Health and Outdoor Play Spaces. *International journal of environmental research and public health,* 16.

PERRY, C. & HOFFMAN, B. 2010. Assessing tribal youth physical activity and programming using a community-based participatory research approach. *Public Health Nurs,* 27**,** 104-14.

PERRY, S., CARPENTER, S. & IDEAS, C. 2016. Preliminary development and piloting of a user-generated routine outcome measure in a children and young people's counselling service. *Counselling and Psychotherapy Research,* 16**,** 171-182.

PETTEWAY, R. J., SHEIKHATTARI, P. & WAGNER, F. 2019. Toward an Intergenerational Model for Tobacco-Focused CBPR: Integrating Youth Perspectives via Photovoice. *Health promotion practice,* 20**,** 67-77.

PHILLIPS, B., DEPANI, S. & MORGAN, J. 2019. What do families want to improve in the management of paediatric febrile neutropenia during anti-cancer treatment? Report of a patient/public involvement group. *BMJ paediatrics open,* 3**,** e000398.

PINSKER, E. A., CALL, K. T., TANAKA, A., KAHIN, A. A., DAR, S. I., GANEY, A., DUBOIS, D. K. & OKUYEMI, K. S. 2017. The Development of Culturally Appropriate Tobacco Prevention Videos Targeted Toward Somali Youth. *Prog Community Health Partnersh,* 11**,** 129-136.

REED, L. A., SHARKEY, J. D. & WROBLEWSKI, A. 2021. Elevating the Voices of Girls in Custody for Improved Treatment and Systemic Change in the Juvenile Justice System. *American Journal of Community Psychology,* 67**,** 50-63.

RODRIGUEZ, E. M., GULBAS, L. E., GEORGE-JONES, J., LEIJA, A., BURROWS, D. & NEAVEL, C. 2019. Interdisciplinary perspectives on an integrated behavioral health model of psychiatry in pediatric primary care: A community-based participatory research study. *Community Mental Health Journal,* 55**,** 569-577.

ROHIT, A., MCCARTHY, L., MACK, S., KIRKHAM, R., SILVER, B., TURNER, S., BOFFA, J., BAUR, L. A., CANUTO, K., DABELEA, D., SAUDER, K. A. & MAPLE-BROWN, L. 2021. The adaptation of a youth diabetes prevention program for aboriginal children in central Australia: Community perspectives. *International Journal of Environmental Research and Public Health,* 18**,** 9173.

ROWE, S. C., DAVENPORT, T. A., EASTON, M. A., JACKSON, T. A., MELSNESS, J., OTTAVIO, A., SINCLAIR, J. & HICKIE, I. B. 2020. Co-designing the InnoWell Platform to deliver the right mental health care first time to regional youth. *Australian Journal of Rural Health,* 28**,** 190-194.

RUSSELL, C., NEUFELD, M., SABIONI, P., VARATHARAJAN, T., ALI, F., MILES, S., HENDERSON, J., FISCHER, B. & REHM, J. 2019. Assessing service and treatment needs and barriers of youth who use illicit and non-medical prescription drugs in Northern Ontario, Canada. *PloS one,* 14**,** e0225548.

RUSSETTE, H. C., SEMMENS, E. O., GRAHAM, N. & SWANSON, S. 2021. Relationship-building to develop an Indigenous community-based epidemiological study investigating developmental resilience factors among children with prenatal substance exposure. *Journal of Ethnicity in Substance Abuse*.

SADIKOVA, E., MEAGHER, H., GOHARI, D., KIRBY, L., RUSSELL, L., POWERS, M. D., STRANG, J. F., WILLING, L., KNAUSS, M., VAN DER MIESEN, A., MCGUIRE, J. K., KENWORTHY, L., CAPLAN, R., PERVEZ, N., FREEMAN, A., ZAKS, Z., BALLEUR, A., ROWLANDS, D. W., SIBARIUM, E., MCCOOL, M. A., EHRBAR, R. D., WYSS, S. E., WIMMS, H., TOBING, J., THOMAS, J., AUSTEN, J., PINE, E., GRIFFIN, A. D., JANSSEN, A., GOMEZ-LOBO, V., BRANDT, A., MORGAN, C. & ANTHONY, L. G. 2020. A Clinical Program for Transgender and Gender-Diverse Neurodiverse/Autistic Adolescents Developed through Community-Based Participatory Design. *Journal of clinical child and adolescent psychology : the official journal for the Society of Clinical Child and Adolescent Psychology, American Psychological Association, Division 53***,** 1-16.

SALAMI, B., DENGA, B., TAYLOR, R., AJAYI, N., JACKSON, M., ASEFAW, M. & SALMA, J. 2021. Access to mental health for Black youths in Alberta. *L'acces des jeunes Noirs de l'Alberta aux services en sante mentale.,* 41**,** 245-253.

SALERNO VALDEZ, E., KORCHMAROS, J., SABO, S., GARCIA, D. O., CARVAJAL, S. & STEVENS, S. 2019. How the U.S.-Mexico border influences adolescent substance use: Youth participatory action research using photovoice. *International Journal of Drug Policy,* 73**,** 146-155.

SALINAS-MIRANDA, A. A., KING, L. M., SALIHU, H. M., WILSON, R. E., NASH, S., COLLINS, S. L., BERRY, E. L., AUSTIN, D., SCARBOROUGH, K., BEST, E., COX, L., KING, G., HEPBURN, C., BURPEE, C., BRISCOE, R. & BALDWIN, J. 2020. Protective Factors Using the Life Course Perspective in Maternal and Child Health. *Engage!,* 1**,** 69-86.

SAMIR, N., DIAZ, A. M., HODGINS, M., MATIC, S., BAWDEN, S., KHOURY, J., EAPEN, V. & LINGAM, R. 2021. Speaking Softly and Listening Hard: The Process of Involving Young Voices from a Culturally and Linguistically Diverse School in Child Health Research. *International journal of environmental research and public health,* 18.

SANDERS, C. & BRAY, L. 2014. Examining professionals' and parents' views of using transanal irrigation with children: Understanding their experiences to develop a shared health resource for education and practise. *J Child Health Care,* 18**,** 145-55.

SANGALANG, C. C., NGOUY, S. & LAU, A. S. 2015. Using community-based participatory research to identify health issues for Cambodian American youth. *Fam Community Health,* 38**,** 55-65.

SCHAPIRO, N. A., GREEN, E. K., KALLER, S., BRINDIS, C. D., RODRIGUEZ, A., ALKEBULAN-ABAKAH, M. & CHEN, J.-L. 2021. Impact on Healthy Behaviors of Group Obesity Management Visits in Middle School Health Centers. *Journal of School Nursing,* 37**,** 87-98.

SCHIPPKE, J., PROVVIDENZA, C. & KINGSNORTH, S. 2017. Peer support for families of children with complex needs: Development and dissemination of a best practice toolkit. *Child Care Health Dev,* 43**,** 823-830.

SCHWARTZ, A. E. & KRAMER, J. M. 2021. Inclusive approaches to developing content valid patient-reported outcome measure response scales for youth with intellectual/developmental disabilities. *British Journal of Learning Disabilities,* 49**,** 100-110.

SERDA, B.-C., PLANAS-LLADO, A., SOLER-MASO, P. & DELVALLE, A. 2021. Health promotion in secondary schools: participatory process for constructing a self-assessment tool. *Health promotion international*.

SETTHEEKUL, S., FONGKAEW, W., VISESKUL, N., BOONCHIENG, W. & VOSS, J. G. 2019. Factors influencing sexual risk behaviors among adolescents: A community-based participatory study. *Nursing & health sciences,* 21**,** 186-197.

SHAIKH, M., BEAN, C., BERGHOLZ, L., ROJAS, M., ALI, M. & FORNERIS, T. 2021. Integrating a Sport-Based Trauma-Sensitive Program in a National Youth-Serving Organization. *Child & Adolescent Social Work Journal,* 38**,** 449-461.

SIMONDS, V. W., KIM, F. L., LAVEAUX, D., PICKETT, V., MILAKOVICH, J. & CUMMINS, J. 2019. Guardians of the Living Water: Using a Health Literacy Framework to Evaluate a Child as Change Agent Intervention. *Health Education & Behavior,* 46**,** 349-359.

SINKO, L., BECK, D. & SENG, J. 2020. Developing the TIC Grade: A Youth Self-Report Measure of Perceptions of Trauma-Informed Care. *Journal of the American Psychiatric Nurses Association***,** 1078390320970652.

SIRDENIS, T. K., HARPER, G. W., CARRILLO, M. D., JADWIN-CAKMAK, L., LOVELUCK, J., PINGEL, E. S., BENTON, A., PETERSON, A., POLLARD, R., BAUERMEISTER, J. A. & KEGLER, M. C. 2019. Toward Sexual Health Equity for Gay, Bisexual, and Transgender Youth: An Intergenerational, Collaborative, Multisector Partnerships Approach to Structural Change. *Health Education & Behavior,* 46**,** 88S-99S.

SLIGO, J., JONES, B., DAVIES, C., EGAN, R., INGHAM, T., HANCOX, R. J. & RICHARDS, R. 2019. The experiences of young people with chronic illness in New Zealand: A qualitative study. *Child: Care, Health & Development,* 45**,** 660-669.

SLINING, M., WILLS, S., FAIR, M., STEPHENSON, J., KNOBEL, S., PEARSON, M., PROSTKO, T., SMYERS, J., TIMBERLAKE, J. & NEGRETE, M. 2021. LiveWell in early childhood: results from a two-year pilot intervention to improve nutrition and physical activity policies, systems and environments among early childhood education programs in South Carolina. *BMC Public Health,* 21**,** 1-9.

SMITS, R. M., VELDHUIJZEN, D. S., VAN MIDDENDORP, H., HISSINK MULLER, P. C. E., ARMBRUST, W., LEGGER, E., WULFFRAAT, N. M. & EVERS, A. W. M. 2020. Pharmacological conditioning for juvenile idiopathic arthritis: a potential solution to reduce methotrexate intolerance. *Pediatric rheumatology online journal,* 18**,** 12.

STALBERG, A. 2021. Design and redesign of the IACTA app, an interactive communication tool intended to facilitate young children's participation in healthcare situations. *Journal of pediatric nursing,* 61**,** 260-268.

STOCK, N. M., HAMMOND, V., HEARST, D., OWEN, T., EDWARDS, Z., RIDLEY, M. & RUMSEY, N. 2020. Achieving Consensus in the Measurement of Psychological Adjustment to Cleft Lip and/or Palate at Age 8+ Years. *Cleft Palate-Craniofacial Journal,* 57**,** 746-752.

SURACHAI, C., WARUNEE, F., STONE, T. E., HUNSA, S. & SUMALEE, L. 2019. Development and Evaluation of a Suicide Prevention Program for Secondary School Students. *Pacific Rim International Journal of Nursing Research,* 23**,** 201-213.

SUTTON, J., HUWS, J. & BURTON, C. 2021. A sleep hygiene tool for children with developmental disabilities. *Nursing Times,* 117**,** 32-36.

SUTTON, J. E., HUWS, J. C. & BURTON, C. R. 2020. Sleep hygiene education and children with developmental disabilities: Findings from a co-design study. *Journal of Intellectual Disabilities,* 24**,** 522-542.

TAPERA, T., WILLIS, N., MADZEKE, K., NAPEI, T., MAWODZEKE, M., CHAMOKO, S., MUTSINZE, A., ZVIRAWA, T., DUPWA, B., MANGOMBE, A., CHIMWAZA, A., MAKONI, T. M., MANDEWO, W., SENKORO, M., OWITI, P., TRIPATHY, J. P. & KUMAR, A. M. V. 2019. Effects of a Peer-Led Intervention on HIV Care Continuum Outcomes Among Contacts of Children, Adolescents, and Young Adults Living With HIV in Zimbabwe. *Global health, science and practice,* 7**,** 575-584.

TEBB, K. P., LENG TRIEU, S., RICO, R., RENTERIA, R., RODRIGUEZ, F. & PUFFER, M. 2019. A Mobile Health Contraception Decision Support Intervention for Latina Adolescents: Implementation Evaluation for Use in School-Based Health Centers. *JMIR mHealth and uHealth,* 7**,** e11163.

TESFAYE, R., COURCHESNE, V., YUSUF, A., SAVION-LEMIEUX, T., SHIKAKO-THOMAS, K., ELSABBAGH, M., SINGH, I., MIRENDA, P., KERNS, C., ZAIDMAN-ZAIT, A., WADDELL, C., SMITH, I. M., NICHOLAS, D., SZATMARI, P., BENNETT, T., DUKU, E., GEORGIADES, S., VAILLANCOURT, T. & ZWAIGENBAUM, L. 2019. Assuming ability of youth with autism: Synthesis of methods capturing the first-person perspectives of children and youth with disabilities. *Autism,* 23**,** 1882-1896.

THOMPSON, J., SPENCER, G. & CURTIS, P. 2021. Children's perspectives and experiences of the COVID-19 pandemic and UK public health measures. *Health expectations : an international journal of public participation in health care and health policy*.

THORBURN, B., GAVEY, N., SINGLE, G., WECH, A., CALDER-DAWE, O. & BENTON-GREIG, P. 2021. To send or not to send nudes: New Zealand girls critically discuss the contradictory gendered pressures of teenage sexting. *Women's Studies International Forum,* 85.

TIWARI, T., SHARMA, T., HARPER, M., ZACHER, T., ROAN, R., GEORGE, C., SWYERS, E., TOLEDO, N., BATLINER, T., BRAUN, P. A. & ALBINO, J. 2015. Community Based Participatory Research to Reduce Oral Health Disparities in American Indian Children. *Journal of family medicine,* 2**,** 1028.

TOWNSON, J., GREGORY, J. W., COWLEY, L., GALLAGHER, D., CHANNON, S., ROBLING, M., WILLIAMS, D., HUGHES, C., MURPHY, S. & LOWES, L. 2017. Establishing the feasibility of a community and primary health care intervention to raise awareness of symptoms of Type 1 Diabetes-The Early Detection of Type 1 Diabetes in Youth (EDDY) study. *Pediatr Diabetes,* 18**,** 955-963.

UCHIMA, O. K., GARCIA, B. K., AGUSTIN, M. L. & OKIHIRO, M. M. 2021. Insights in Public Health: Ask the Keiki: Perceived Factors that Affect Asthma Among Adolescents from the Wai'anae Coast Using Photovoice. *Hawai'i journal of health & social welfare,* 80**,** 222-229.

UDING, N., SETY, M. & KIECKHEFER, G. M. 2007. Family involvement in health care research: The "Building on Family Strengths" case study. *Families, Systems, & Health,* 25**,** 307-322.

VALDEZ, E. S., ANDRADE, R. & PALAFOX, M. M. 2019. Developing a Binational Community-Based Participatory Research Partnership to Address Reproductive Health on the U.S.-Mexico Border. *Progress in community health partnerships : research, education, and action,* 13**,** 265-271.

VALDEZ, E. S., VALDEZ, L. & GARCIA, D. O. 2021. Using Participatory Methods to Enhance Youth Engagement in Substance Use Research. *Health promotion practice***,** 1524839921990005.

VALENTINE, S. E., AHLES, E. M., SILVA, L. E. D. D., PATRICK, K. A., BALDWIN, M., CHABLANI-MEDLEY, A., SHTASEL, D. L. & MARQUES, L. 2019. Community-Based Implementation of a

Paraprofessional-Delivered Cognitive Behavioral Therapy Program for Youth Involved with the Criminal Justice System. *Journal of Health Care for the Poor & Underserved,* 30**,** 841-865.

VAN GALEN, K., FISCHER, K., MAUSER-BUNSCHOTEN, E., LAVIN, M., SKOUW-RASMUSSEN, N., NOONE, D., POLLARD, D., GOMEZ, K., KHAIR, K., VAN LOON, E., BAGOT, C. N., ELFVINGE, P., D'OIRON, R. & ABDUL-KADIR, R. 2021. European principles of care for women and girls with inherited bleeding disorders. *Haemophilia,* 27**,** 837-847.

VAN NULAND, A., REDDY, T., QUASSEM, F., VASSALLI, J.-D. & BERG, A. T. 2021. PACS1-Neurodevelopmental disorder: clinical features and trial readiness. *Orphanet Journal of Rare Diseases,* 16**,** 1-10.

VAN STAA, A., JEDELOO, S., LATOUR, J. M. & TRAPPENBURG, M. J. 2010. Exciting but exhausting: experiences with participatory research with chronically ill adolescents. *Health Expect,* 13**,** 95-107.

VANDENBERG, L. N., KOLLA, S., LAPLANTE, C. D. & JERRY, D. J. 2020. The Mouse Mammary Gland: a Tool to Inform Adolescents About Environmental Causes of Breast Cancer. *Journal of cancer education : the official journal of the American Association for Cancer Education,* 35**,** 1094-1100.

VEATUPU, L., PULOKA, V., SMITH, M., MCKERCHAR, C. & SIGNAL, L. 2019. Me'akai in Tonga: Exploring the nature and context of the food Tongan children eat in ha'apai using wearable cameras. *International Journal of Environmental Research and Public Health,* 16**,** 1681.

WALKER, A., COLQUITT, G., ELLIOTT, S., EMTER, M. & LI, L. 2020. Using participatory action research to examine barriers and facilitators to physical activity among rural adolescents with cerebral palsy. *Disability & Rehabilitation,* 42**,** 3838-3849.

WALKER, E., SHAW, E., NUNNS, M., MOORE, D. & THOMPSON COON, J. 2020. No evidence synthesis about me without me: Involving young people in the conduct and dissemination of a complex evidence synthesis. *Health Expectations: An International Journal of Public Participation in Health Care & Health Policy***,** No-Specified.

WAMBUA, G. N., MUSINDO, O., MACHUKA, J. & KUMAR, M. 2019. Engagement and partnership with peer mentors in the development of the "Positive and Healthy Living Program": a process paper. *AIDS Care,* 31**,** 1145-1151.

WATERLANDER, W. E., PINZON, A. L., VRIJKOTTE, T., STRONKS, K., BUSCH, V., VAN HOUTUM, L., VAN DE VLASAKKER, S., VERHOEFF, A., DEN HERTOG, K., ALTENBURG, T., SINGH, A., ANSELMA, M., EMKE, H., CHINAPAW, M., DIJKSTRA, C., HALBERSTADT, J., RENDERS, C., SEIDELL, J., HERMANS, R., OVERMAN, M., KREMERS, S. P. J., VAN DEN EYNDE, E., VAN DEN AKKER, E. & NUSSELDER, W. J. 2020. A system dynamics and participatory action research approach to promote healthy living and a healthy weight among 10-14-year-old adolescents in Amsterdam: The LIKE programme. *International Journal of Environmental Research and Public Health,* 17**,** 1-18.

WATSON-THOMPSON, J., JESSOP, N., HASSABALLA, I., VANCHY, P., HENDERSON, J. & MOORE, C. 2020. Together Helping Reduce Youth Violence for Equity (ThrYve): Examining the Development of a Comprehensive Multisectoral Approach to Youth Violence Prevention. *American Journal of Community Psychology,* 66**,** 244-255.

WEBER, A. M., VOOS, K. C., BAKAS, T. M., RICE, J. B., BLATZ, M. A., RIBEIRO, A. P. D., TUBBS-COOLEY, H. L., ROTA, M. J. & KAPLAN, H. C. 2021. A clinical-academic partnership to develop a family management intervention for parents of preterm infants. *Journal of clinical nursing*.

WELCH, M. L., MULCAHY, A., BEVERLY, J. G., MCMAUGHAN, D. J., OZMETIN, J. P., IMANPOUR, S. & NAISER, E. 2021. Framing the front door: co-creating a home health care assessment of service need for children with disabilities. *Home health care services quarterly,* 40**,** 231-246.

WILLIS, E., GUNDACKER, C., HARRIS, M. & MAMELEDZIJA, M. 2020. Improving Immunization and Health Literacy Through a Community-Based Approach. *Studies in Health Technology & Informatics,* 269**,** 142-152.

WOGRIN, C., LANGHAUG, L. F., MARUVA, C. & WILLIS, N. 2019. Development and piloting of a novel, peer-led bereavement intervention for young people living with HIV in Zimbabwe. *Journal of Child & Adolescent Mental Health,* 31**,** 13-24.

WOLFE, J., BLUEBOND-LANGNER, M., HEIN, K., KNOCHEL, K., ZAIMOVIC, V., REIMANN, D., MONZ, A., HEITKAMP, N., BORASIO, G. D. & FÜHRER, M. 2020. Identifying key elements for paediatric advance care planning with parents, healthcare providers and stakeholders: A qualitative study. *Palliative Medicine,* 34**,** 300-308.

WOLFSON, M., WAGONER, K. G., RHODES, S. D., EGAN, K. L., SPARKS, M., ELLERBEE, D., SONG, E. Y., DEBINSKI, B., TERRILLION, A., VINING, J. & YANG, E. 2017. Coproduction of Research Questions and Research Evidence in Public Health: The Study to Prevent Teen Drinking Parties. *Biomed Res Int,* 2017**,** 3639596.

YOUNG, J., WATSON, K., CRAIGIE, L., NEVILLE, J. & HUNT, J. 2019. Best practice principles for research with Aboriginal and Torres Strait Islander communities in action: Case study of a safe infant sleep strategy. *Women & Birth,* 32**,** 460-465.

YOUNG, N., WABANO, M. J., BLIGHT, S., BAKER-ANDERSON, K., BEAUDIN, R., MCGREGOR, L. F., MCGREGOR, L. & BURKE, T. 2017. Relevance of the Aboriginal Children's Health and Well-being Measure Beyond Wiikwemkoong. *Rural and Remote Health,* 17.

ZAHND, W. E., SMITH, T., RYHERD, S. J., CLEER, M., ROGERS, V. & STEWARD, D. E. 2017. Implementing a Nutrition and Physical Activity Curriculum in Head Start Through an Academic-Community Partnership. *J Sch Health,* 87**,** 465-473.

ZEITLIN, J., SENTENAC, M., MORGAN, A. S., ANCEL, P. Y., BARROS, H., CUTTINI, M., DRAPER, E., JOHNSON, S., LEBEER, J., MAIER, R. F., NORMAN, M. & VARENDI, H. 2020. Priorities for

collaborative research using very preterm birth cohorts. *Archives of Disease in Childhood: Fetal and Neonatal Edition,* 105**,** 538-544.

ZELENKO, O., CHEERS, J., STOYANOV, S. R., STANEVA, A., KAVANAGH, D. J., SMITH, C., SADE, G. & HIDES, L. 2021. Development of the Niggle App for Supporting Young People on Their Dynamic Journey to Well-being: Co-design and Qualitative Research Study. *JMIR mHealth and uHealth,* 9**,** e21085.

ZLOTNICK, C., GOLDBLATT, H., BIRENBAUM-CARMELI, D., DISHON, Y., TAYCHAW, O. & SHADMI, E. 2019. The impact of adolescents' racial and ethnic self-identity on hope. *Health & social care in the community,* 27**,** e705-e715.

ZOELLNER, J. M., YOU, W., HILL, J. L., BROCK, D.-J. P., YUHAS, M., ALEXANDER, R. C., PRICE, B. & ESTABROOKS, P. A. 2019. A comparative effectiveness trial of two family-based childhood obesity treatment programs in a medically underserved region: Rationale, design & methods. *Contemporary clinical trials,* 84**,** 105801.

ZUNIGA, C., WOLLUM, A., KATCHER, T. & GRINDLAY, K. 2019. Youth Perspectives on Pharmacists' Provision of Birth Control: Findings From a Focus Group Study. *Journal of Adolescent Health,* 65**,** 514-519.
